# The association of social networks and depression in community-dwelling older adults: a systematic review

**DOI:** 10.1186/s13643-024-02581-6

**Published:** 2024-06-20

**Authors:** Amelie Reiner, Paula Steinhoff

**Affiliations:** Institute of Sociology and Social Psychology, University of Cologne, Albertus-Magnus-Platz, 50923 Cologne, Germany

**Keywords:** Social network, Mental health, Depression, Older adults, Systematic review

## Abstract

**Background and objective:**

Depression is a globally prevalent mental condition, particularly among older adults. Previous research has identified that social networks have a buffering effect on depression. Existing systematic reviews have either limited their research to specific geographic areas or provided evidence from over a decade ago. The vast body of recent literature particularly from the last decade emphasizes the need for a comprehensive review. This systematic review aims to analyze the association of structural aspects of social networks and depression in older adults.

**Methods:**

The electronic databases APA PsycINFO, ProQuest, PSYINDEX, PubMed, Scopus, SocINDEX, and Web of Science were searched from date of data base inception until 11 July 2023. Studies were eligible for inclusion if they reported on community-dwelling older adults (defined as a mean age of at least 60 years old), had an acceptable definition for depression, referred to the term social network in the abstract, and were published in English. Quality was appraised using the Newcastle Ottawa Scale for cross-sectional and longitudinal studies. Outcome data were extracted independently from each study and analyzed by direction of the relationship, social network domain and cross-sectional or longitudinal study design.

**Results:**

In total, 127 studies were included. The study categorizes structural network aspects into seven domains and finds that larger and more diverse networks, along with closer social ties, help mitigate depression. The literature on the relationships between depression and network density, homogeneity, and geographical proximity is scarce and inconclusive.

**Discussion and implications:**

Despite inconsistent findings, this review highlights the importance of quantifying complex social relations of older adults. Limitations of this review include publication and language bias as well as the exclusion of qualitative research. Further research should use longitudinal approaches to further investigate the reciprocal relationship between social networks and depression. Following this review, interventions should promote the integration of older adults in larger and more diverse social settings.

Other: This work was supported by the Deutsche Forschungsgemeinschaft (DFG, German Research Foundation) under Grant [454899704]. This systematic review was pre-registered. The review-protocol can be accessed at https://doi.org/10.17605/OSF.IO/6QDPK.

**Supplementary Information:**

The online version contains supplementary material available at 10.1186/s13643-024-02581-6.

## Background and objective

Depression is a mental condition that is particularly prevalent among older adults [1]. Scholars have identified a significant association between social networks and depression, with socially integrated older adults showing lower levels of depression than less socially integrated older adults [2, 3]. As older adults face a decreasing number of social relationships and a shrinking social network over their life course [4], this growing population is at risk for depression. Systematizing and quantifying the social networks of older adults is vital to understanding their relationship with depression. The prevalence of depression will increase in the future. Understanding the aspects of social networks that are particularly important for preventing depressive symptomatology in older adults will allow appropriate social gerontological interventions.

Previous systematic reviews have generated important insights into the relationship between social networks and mental health. Across several geographical areas, various social network measures have been found to be significantly associated with mental health in older adults (Middle Eastern countries: [5]; Iran: [6]), and specifically depression (Asia: [2]; Western countries: [7]). However, only one systematic review has addressed the relationship between social networks and depression among older adults without restricting its evidence to a geographical area [3]. While Schwarzbach et al.’s [3] review has been helpful, new evidence about the social relations of older adults and depression outcomes must be reviewed because a significant amount has emerged over the last decade.

Additionally, previous studies and literature reviews have loosely applied the concept of social networks and engaged with different definitions and measures of social networks [8, 9]. A social network is traditionally defined as the quantifiable ties binding individuals, families, communities, or businesses (i.e., nodes) together through a shared need, aim, or interest [10, 11]. The nature of one’s social network was found to have a significant influence on an individual’s life expectancy, mortality rate, quality of life, and health-related behaviors [8]. Generally, the literature has distinguished between the quantitative/structural and qualitative/functional aspects of social relationships [12, 13]. Qualitative aspects refer to the social network’s function, including the potential of social relationships, such as social support, the perceived quality of support provided, relationship satisfaction, loneliness and social isolation [13, 14]. In contrast, quantitative aspects refer to the network’s structure, including its size, composition, and the frequency of contact between network members. Recently, it has become increasingly clear that quantifying social networks, which provides an objective measure of the structure of relationships, is particularly suited for understanding their association with critical health outcomes, such as cognitive decline [14], dementia [15], and mortality [16]. As structural aspects of social networks are causally prior to functional aspects, this review exclusively focuses on their structural aspects while examining their relationship with depression in older adults.

The relationship between social networks and depression can be considered reciprocal. The main effect model [17] states that social networks positively affect psychological state through mechanisms such as social recognition, a sense of belonging, and normative guidance for health-promoting behavior. Conversely, depression may affect the extent of social networks by causing social withdrawal and decreased social participation. Older adults who experience depression in later life often struggle with maintaining larger and more diverse personal networks and experience disruptions in their contact with social network members [18]. Existing research has predominantly focused on the effect of social networks on depression. Conversely, the reversed effect of depression on social networks has been largely neglected [19, 20].

This systematic review, therefore, aims to synthesize the evidence about the relationship between structural aspects of social networks and depression in community-dwelling older adults. It addresses two research questions: (1) How do structural aspects of social networks impact depression outcomes in community-dwelling older adults? (2) How does depression impact structural aspects of social networks of community-dwelling older adults? It strives to provide a comprehensive picture by gathering cross-sectional as well as longitudinal evidence and by focusing on the reciprocal relationship between social networks and depression in older adults.

## Methods

This systematic review was pre-registered. The review-protocol can be accessed at 10.17605/OSF.IO/6QDPK. In addition, we followed PRISMA guidelines for the reporting of this systematic review ([21]; see Additional file 1, Table A1).

### Eligibility criteria

We expected to include peer-reviewed articles on the association of structural social network characteristics and depression among community-dwelling older adults. Following the World Health Organization (WHO; [22]), we define older adults as those, being 60 years and older. To counteract possible regional selection bias induced by language knowledge, we focused on English publications only. We did not exclude studies based on publication year or geographic area.

Related previous systematic reviews informed the inclusion and exclusion criteria [2, 3, 5–8, 13, 23–25]. Articles were included if the population of interest consisted of community-dwelling adults, specifically those older than 40 years, with a study mean age of at least 60 years. We opted for a minimum age in order to include relevant age studies from the age of 40 (e.g., the German DEAS), but focused on older adults by deciding that the mean age of the study participants must be at least 60 years, following the definition of older adults. The exposure or outcome focused on social networks, explicitly mentioned in the abstract of the studies. Further exposure or outcome of interest was depression, with an acceptable definition involving diagnostic criteria or a cut-off point on a depression rating scale. The association between social networks and depression had to be reported using a multivariate analysis adjusting for any confounders (the specifics of the included confounders are evaluated in the quality assessment). Only peer-reviewed journal articles published in English were considered for inclusion. Articles were excluded if they focused on patient groups or included institutionalized individuals, unless the analyses separated community-dwelling and institutionalized participants. Additionally, studies were excluded if they referred to recalled social network characteristics from the past, such as youth and adolescence, to measure present depression outcomes, or if they exclusively focused on online social networks. In terms of study types, editorials, study protocols, conference proceedings, comments, reviews, qualitative studies, grey literature, case studies, and intervention studies were excluded. An overview of the studies that appeared to meet the inclusion criteria but were ultimately excluded and the reasons for this can be found in the Additional file 1, Table A2.

### Search strategy

The systematic database search was performed from date of data base inception up to 11 July 2023. The keywords used for the search strategy included related terms for: “depression” AND “social networks” AND “older adults” (see pre-registered review protocol). These were informed by related systematic reviews about the three main terms [2, 3, 5–8, 13, 23–25]. The following seven databases were searched using the same keywords and search designs: APA PsycINFO, ProQuest, PSYINDEX, PubMed, Scopus, SocINDEX, and Web of Science. We also conducted manual searches for potentially eligible studies from reference lists of related systematic reviews [2, 3, 5–8, 13, 23–25].

### Study selection

References from the seven databases were imported into Rayyan [26]. After deduplication, two researchers (AR, PS) independently screened titles and abstracts, forwarding potentially eligible papers for full text review. Two researchers (AR, PS) independently assessed the full text of potentially eligible citations against the eligibility criteria. Disagreements and discrepancies were resolved by consensus between the researchers. The study selection process was piloted twice with a random sample of a hundred studies of the overall sample per pilot. Piloting the study selection process improves the reliability and validity of the review by ensuring all reviewers have a clear and consistent understanding of the selection process [27].

### Data extraction

Using a standardized data collection form informed by related reviews [2, 3, 5–8, 13, 23–25], two reviewers (AR, AL) independently extracted data on the study population including their sample size, average age and age range, gender ratio, and country. Further, we extracted information on the measurement of depression, the social network assessment, type of social ties, potential exclusion of population groups, data source, the statistical methods, and the results. The outcomes of interest were structural aspects of social networks and/or depression scores among community-dwelling older adults. Any disagreements were resolved by discussion. If this failed, a third reviewer (PS) was consulted. The data extraction process was piloted once with a random sample of twenty studies to ensure the completeness of all relevant information in the data collection form [28].

### Quality appraisal

Quality was assessed using the Newcastle Ottawa Scale (NOS; [29]) for cross-sectional and longitudinal studies by one reviewer (AR) and double-checked by another reviewer (PS). The NOS has been used in systematic reviews before [2, 30–32]. The NOS awards each article an amount of stars within three domains, with a greater number of stars indicate a higher‐quality study [29]. The study quality is evaluated in terms of design, participant selection, comparability and assessment of exposure and outcome. Following the approach of several reviews [2, 31, 32], we adopted a rigorous methodology to assess the quality of studies, adhering to predetermined thresholds for converting the NOS to Agency for Health Research and Quality (AHRQ) standards. For a cross-sectional study to be considered of good quality, it needed to attain between 3 and 5 stars in the selection domain, alongside 1 or 2 stars in the comparability domain, and finally, 2 or 3 stars in the outcome domain. Those studies that achieved 2 stars in the selection domain, coupled with 1 or 2 stars in comparability, and 2 or 3 stars in outcome were classified as fair quality. However, studies falling short of these criteria were deemed poor quality; they either obtained 0 or 1 star in the selection domain, 0 stars in comparability, or 0 or 1 stars in outcome. In contrast, a longitudinal study was considered of good quality if it garnered between 3 and 4 stars in the selection domain, along with 1 or 2 stars in the comparability domain, and finally, 2 or 3 stars in the outcome domain. Those longitudinal studies achieving 2 stars in the selection domain, paired with 1 or 2 stars in comparability, and 2 or 3 stars in outcome were categorized as fair quality. Conversely, studies failing to meet these benchmarks were classified as poor quality; they either received 0 or 1 star in the selection domain, 0 stars in comparability, or 0 or 1 stars in outcome. For the analyses, we included all studies irrespective of the quality assessment results. However, when excluding studies which were considered as poor quality in a sensitivity analysis, the results were found to remain largely stable.

### Synthesis method

Citations were firstly sub-grouped by direction of the relationship, then by structural aspect of social networks, and afterwards by the cross-sectional or longitudinal study design. In a further step, we count the significant associations against the insignificant associations. We compare the significant results across study design to identify differences between cross-sectional and longitudinal relationships. Further, we compare the effects of interest across structural aspects of social networks in the discussion. Tables are used to display the sub-grouped evidence. Further comparisons were carried out by geographical location, gender, family versus friends’ social ties and functional versus structural social network aspects. Findings are reported narratively.

## Results

### Sample description

**Fig.1:**
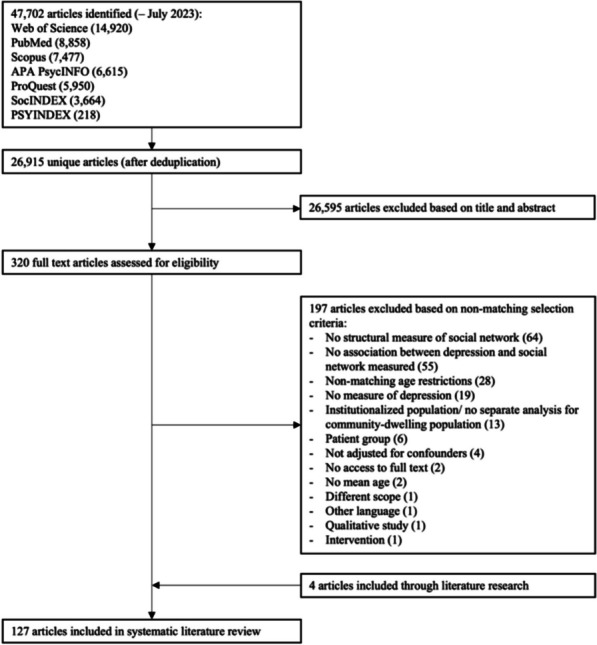
HYPERLINK "sps:id::fig1||locator::gr1||MediaObject::0"Selection flowchart for papers included in the systematic review

Starting from an initial result of 47,702 entries, 26,915 unique citations were identified. The two authors (AR, PS) independently screened the titles and abstracts, resulting in 320 potentially eligible articles. Any disagreement over the eligibility of individual studies was resolved through discussion. After adhering to strict inclusion and exclusion criteria, 127 unique publications were identified. Figure 1 Visualizes a PRISMA flowchart of the selection process.

The quality appraisal for each NOS-domain and overall evaluation can be found in the Additional file 1, Table A3 for cross-sectional studies and Table A4 for longitudinal studies. Two thirds of the studies (*n* = 86) were classified as good-quality studies, 27 articles with fair quality and 15 articles with poor quality.

The included articles were published between 1985 and 2023, with half published later than 2016. This highlights the vast body of research that has been conducted on this association, particularly in the last decade. The range of sample sizes was 53 to 60,918, with a median sample size of 1349 respondents. The geographic location of most of the studies was North America (*n* = 46), followed by Asian countries (*n* = 42). Thirty-four studies were conducted in European countries (and Israel), and only three were conducted in South American countries. One study has a mixed geographical location by comparing older adults in North America to those in Asia [33]. One study did not specify its geographic location [34].

The majority of studies made use of validated instruments to assess particularly depression. They either used various forms of the Center for Epidemiologic Studies Depression Scale (CES-D, *n* = 58) or the Geriatric Depression Scale (GDS, *n* = 42) to assess depression. Other studies used the EURO-D scale (*n* = 12), the Composite International Diagnostic Interview (CIDI, *n* = 3), the nine-item Patient Health Questionnaire (PHQ-9, *n* = 3), or other validated instruments (*n* = 9).

Most studies focused on the cross-sectional relationship between the social networks of older adults and depression (*n* = 96), while 30 articles examined the relationship longitudinally. Only one article had both a cross-sectional and longitudinal focus [35]. In most aspects of social networks, there were no apparent differences between the cross-sectional and longitudinal investigations. Additionally, 90% (*n* = 114) of the studies exclusively used depression as an outcome variable, while 6% (*n* = 8) exclusively used social network variables as outcome variables. Only five studies focused on the existence of a bi-directional relationship [19, 20, 36–38].

All risk factors for depression related to social networks used within the studies were categorized. Seven structural aspects of social networks were identified: network composition, contact frequency, network density, homo-/heterogeneity, network size, geographic proximity, and network scales. Table 1 provides an overview of the social network aspect descriptions. Notably, ties to friends and family were the covered most frequently in social network measures. The results were largely stable across geographic areas.

**Table 1:** Description of the structural aspects of social networks

Structural aspect of social networks	Description
Composition	Measures that describe how a network is composed, either through proportions of family/friends or building a network typology
Contact frequency	Frequency of various forms of contact with different social ties
Density	Indices indicating the extent to which a network is loosely connected [39]
Geographic proximity	Travel distance to social ties in km or time
Homogeneity	Indices for the similarity of one’s social ties to one’s own personality [39]
Scales	Scales mainly capture an individual’s marital status, number and frequency of contacts with children, close relatives, close friends, church group membership, and membership in other community organizations [40]
Size	Number of social relations in the individual’s personal network

### Depression as outcome variable

In total, 119 articles examined structural network aspects’ effects on depression. Ninety articles did so cross-sectionally, and 28 articles did so longitudinally. One article focused on the relationship both cross-sectionally and longitudinally [35].

Most publications focused on network scales (*n* = 44), network size (*n* = 44), network composition (*n* = 30), and contact frequency (*n* = 28) as structural network factors determining depression outcomes in older adults. Significantly fewer articles used density (*n* = 4), geographic proximity (*n* = 3), and homogeneity (*n* = 2). The results are presented below according to their frequency.

#### Network scales

Some articles used standardized network scales to examine various aspects of social networks’ effects on depression among older adults. Most articles used (modifications or translations of) the Lubben Social Network Scale (LSNS) or the Social Network Index (SNI), with higher scores indicating greater social engagement.

Most associations (40 out of 60 = 67%) between network scales and depression among older adults were reported to be significant (Table 2). No meaningful difference was identified between cross-sectional and longitudinal studies concerning effect significance or direction. Consistently, scholars found higher scores on social network scales to buffer depression outcomes among older adults. However, different subscales were used to assess family and friends variables. While some studies suggested that family networks were more predictive of depression outcomes in older adults [41–43], Singh et al. [44] indicated the opposite, suggesting that the friend network scale was significantly associated with depression. They found no significant associations in the children, relatives, and confidant network scales.

**Table 2:** Overview of results: network scales and depression

Author	Depression measure	Social network measure	*N* ^a^	Results ^b^	Quality
*Cross-sectional studies*					
Aung et al., 2016 [45]	GDS-30	SNI	435	+	Good
Bae et al., 2020 [46]	GDS-15	NCGG Social Network Scale	2445	+	Good
Boey & Chiu, 2005 [47]	GDS-15	LSNS Family network Friend network	1034	+ 0/ + (significant in older women, but not men)	Good
Chan & Zeng, 2009 [48]	GDS-15	Social Network Scale (SNS) (family network; networks of friends; helping others; confidence in relationships and living arrangements)	1042	+	Good
Chan & Zeng, 2011 [49]	GDS-15	LSNS	839	+	Good
Chan et al., 2011 [50]	CES-D (11)	LSNS (friends and relatives)	4489	+	Good
Chou & Chi, 2001 [51]	CES-D (20)	LSNS	411	+	Good
Fernández & Rosell, 2022 [41]	PHQ-9	LSNS Family Network (subscale) Friend Network (subscale)	2132	+ +	Good
Gao et al., 2022 [42]	CES-D (10)	LSNS Family Network (subscale) Friend Network (subscale) Total	5934	+ + 0	Good
Gu et al., 2023 [52]	GDS-15	LSNS Family Network (subscale) Friend Network (subscale)	824	0/ + (sig. only among rural older adults, but not urban) 0/ + (sig. only among urban older adults, but not rural)	Good
Hamid et al., 2019 [53]	GDS-15	LSNS	594	+	Good
Jang et al., 2002 [54]	GDS-15	LSNS	406	+	Good
Jang et al., 2011 [55]	CES-D (10)	LSNS	230	0	Fair
Jiang et al., 2022 [56]	GDS-15	LSNS	3769	+	Good
Kim & Lee, 2015 [57]	SGDS-K	LSNS Family Network (subscale) Friend Network (subscale)	949	+ +	Good
Kim et al., 2012 [58]	GDS-15	LSNS	210	+	Good
Kim et al., 2015 [59]	GDS-15	LSNS	147	0	Fair
Klug et al., 2014 [60]	GDS-15	SNI (dichotomous measure: 1–2 = low social network; 3–4 = high social network)	969	0	Good
Lee et al., 2017 [61]	GDS-30	LSNS	200	+	Good
Mehrabi & Béland, 2021 [62]	GDS-15	Social contact score: Number of ties, Number of ties seen least once a month, number of ties being close with, number of ties having called at least once a month Friends Children Grandchildren Siblings	1643	0 0 0 0	Fair
Okwumabua et al., 1997 [63]	CES-D (20)	LSNS	110	+	Poor
Palinkas et al., 1990 [64]	BDI (18)	SNI	1615	+	Poor
Park & Roh, 2013 [65]	GDS-30	LSNS	200	+	Good
Park et al., 2013 [66]	GDS-15 (Korean translation)	SNI	674	+	Good
Park et al., 2019 [67]	CES-D (10)	LSNS Family Network (subscale) Friend Network (subscale)	353	0 0	Good
Roh et al., 2015 [68]	GDS-30 Korean Version	LSNS	200	+	Good
Santini et al., 2015 [69]	CES-D (20)	SNI	4988	+	Good
Singh et al., 2016 [44]	CIDI	Social network scale (Summary scores: number of ties, visual contact, non-visual contact) Children Network Relatives Network Friends Network Confidant Network	630	0 0 + 0	Fair
Sugie et al., 2022 [37]	GDS-15	LSNS (dichotomous, scores < 12 limited network)	268	+	Good
Tang & Xie, 2021 [70]	CES-D	LSNS Family Network (subscale) Friend Network (subscale)	2484	+ +	Good
Tang et al., 2020 [71]	CES-D (9)	LSNS Family Network (subscale) Friend Network (subscale)	7662	+ +	Good
Tang et al., 2023 [43]	CES-D	LSNS Family Network (subscale) Friend Network (subscale)	7601	+ +	Good
Tanikaga et al., 2023 [72]	GDS-15	LSNS	74	+	Good
Taylor, 2021 [73]	CES-D (7)	SNI	2323	0	Good
Tsai et al., 2005 [74]	GDS-15	Social support network: number of relatives or friends who would likely contact the elder and by the quantity of contacts (either by phone or in person) during previous week	1200	+	Good
Wee et al., 2014 [75]	GDS-15	LSNS	559	+	Fair
*Longitudinal studies*					
Byers et al., 2012 [76]	GDS-15	LSNS (dichotomized: below the median averaged LSNS = small social network)	7240	+	Good
Domènech-Abella et al., 2019 [20]	CIDI-SF	SNI	5066	+	Good
Förster et al., 2021 [77]	GDS-15	LSNS-6	679	0	Good
Kuchibhatla et al., 2012 [78]	CES-D (20)	Social interaction scale (summary measure of contact frequency with friends and relatives, and membership in social organizations)	3973	0	Good
Ruan et al., 2022 [79]	CES-D (9)	LSNS	4466	+	Good
Santini et al., 2016 [80]	CES-D (20)	SNI	6105	+	Good
Santini et al., 2017 [81]	CES-D (20)	SNI	6098	+	Good
Zhang et al., 2023 [36]	DASS-21 (depression subscale)	LSNS	634	0	Good

^a^*n*: Sample size, baseline sample was used in longitudinal studies

^b^Results: 0 indicates no sig. relationship (*p* ≥ 0.05), + indicates sig. relationship (*p* < 0.05)

Depression measures: *BDI* Beck Depression Inventory, *CES-D* Centre of Epidemiologic Studies Depression Scale, *CIDI-SF* Composite International Diagnostic Interview (Short Form), *DASS-21* Depression Anxiety Stress Scale, *EURO-D* EURO geriatric depression scale, *GDS* Geriatric Depression Scale, *SGDS-K* Geriatric Depression Scale Short Form Korean Version, *PHQ-9* Patient Health Questionnaire

Social network measures: *LSNS* Lubben Social Network Scale, *NCGG Social Network Scale* National Center for Geriatrics and Gerontology Social Network Scale, *SNI* Social Network Index

The results appear to be largely stable across gender. Most of the studies considering gender differences did not find the association of network scales and depression to differ in women and men [43, 50, 60, 66]. The evidence of studies finding gender differences is inconclusive. While two studies found network scales to be only significant associated with depression in men but not women [68, 80], another study found a significant association for the friends’ subscale in women but not men [47]. Conversely, no gender differences were found regarding the family subscale [47].

#### Network size

Network size was the most frequently studied variable besides network scales. In total, 66 measured associations were found in 44 articles (see Table 3). No meaningful difference was identified between cross-sectional and longitudinal studies concerning effect significance or direction. The results were inconclusive: Half of the studies found no significant association, while the other half provided significant evidence for an effect of social network size on depression in older adults. Of the effects significantly associated with depression, 32 of 33 were negative. This suggests that more extensive social networks are associated with lower levels of depression in older adults.

**Table 3:** Overview of results: network size and depression

Author	Depression measure	Social network measure	*N* ^a^	Results ^b^	Quality
*Cross-sectional studies*					
Antonucci et al., 1997 [82]	CES-D	Total Network Size (people who are important to them; network size: 0–3, 4–7, 8 or more people)	3777	+	Good
Becker et al., 2019 [83]	Euro-D	Total Network Size (up to 7 persons)	52,513	+	Poor
Bisconti & Bergeman, 1999 [84]	CES-D (20)	Network size Family (number of family members who are met or talked to on the phone in a typical week) Friends (number of family members who are met or talked to on the phone in a typical week)	232	0 0	Poor
Braam et al., 1997 [85]	CES-D (20)	Total Network Size (Number of people named in the seven categories: persons living in the same household, children and children-in-law, other relatives, neighbors, people with whom one is working or studying, contacts in organizations and other contacts)	2817	+	Good
Cheng et al., 2014 [86]	GDS-4	Total Network Size (Social convoy questionnaire, network members that are important)	273	+	Poor
Chi & Chou, 2001 [87]	CES-D (20)	Relatives/Kin size Number of relatives seen once a month Number of relatives felt close to Number of friends seen once a month Number of friends felt close to	1106	0 0 + 0 +	Good
Cho et al., 2019 [88]	CES-D (10)	Total Network Size (number of close friends and close relatives: 0, 1–2, 3–5, 6–9, 10 +)	2541	0	Good
Domènech-Abella et al., 2017 [89]	CIDI 3.0	Total Network Size (Berkman-Syme Social Network Index)	3535	+	Good
Dorrance Hall et al., 2019 [90]	CES-D (9)	Total Network Size (persons with whom they talk about important matters and regularly interact)	2249	+	Good
Ermer & Proulx, 2022 [91]	CES-D (11)	Total Network Size (Social network roster)	865	0	Fair
Fredriksen-Goldsen et al., 2013 [92]	CES-D (10)	Total Network Size (Interaction with friends, family members, colleagues, and neighbors in a typical month; calculated and summarized by quartiles)	2439	+	Good
Fuller-Iglesias et al., 2008 [93]	CES-D (20)	Total Network Size (Hierarchical mapping technique)	99	+	Poor
Goldberg et al., 1985 [94]	CES-D (20)	Total Network Size (household members, friends, family members outside of the household in touch during 6 months before; household members and up to 10 friends and 10 family members) Number of confidants	1104	0 +	Good
Han et al., 2007 [95]	KDSKA	Family size/network (number of living parents, spouse, children, grandchildren, and other relatives)	205	0	Fair
Harada et al., 2023 [96]	GDS-15	Kin network (number of siblings, cousins, grandchildren or other relatives with whom respondent or respondent’s spouse interacts on a regular basis (except household members) Friends network (number of friends with whom respondent interacts on a regular basis)	739	+ +	Good
Jeon & Lubben, 2016 [97]	CES-D (20)	Relatives/Kin size Non-kin network size (Total number of relatives/non-relatives participants talked to at least once a month)	424	0 0	Fair
Lee & Chou, 2019 [98]	GDS-15	Friendship size Number of children Relatives/Kin size (Number of children, family members, and friends they felt close to)	850	+ 0 +	Good
Lee et al., 1996 [99]	CES-D (20)	Total Network Size (numbers of living parents, children, and friends)	162	+	Poor
Li et al., 2019 [100]	PHQ-9	Total Network Size (up to 5 people with whom they discuss important things)	3157	+	Fair
Litwin & Levinsky, 2023 [101]	Euro-D	Total Network Size (up to 6 persons with whom they discuss personal matters; one additional person who was important for any reason)	35,145	+	Good
Litwin et al., 2015 [102]	Euro-D	Total Network Size (up to 6 persons with whom they discuss personal matters; one additional person who was important for any reason)	25,245	+	Good
Liu et al., 2016 [33]	CES-D (9)	Friendship size/network (friends in local community: none or few, some or quite a few, a lot)	529	+	Poor
Miller & Lago, 1990 [34]	GDS-15	Total Network Size (hierarchical mapping technique)	53	0	Poor
Minicuci et al., 2002 [103]	CES-D (20)	Number of relatives with close contact Number of close friends	2398	0 0	Good
Palinkas et al., 1990 [64]	BDI (18)	Friendship network size Relatives/Kin size	1615	+ 0	Poor
Pavlidis et al., 2023 [104]	Euro-D	Small network (1–2 members) vs. large network (3 + members) (up to 6 persons with whom they discuss personal matters; one additional person who was important for any reason)	60,918	0	Fair
Pilehvari et al., 2023 [105]	CES-D (20)	Number of people in social network	1170	0	Good
Sonnenberg et al., 2013 [106]	CES-D (20)	Total Network Size (people in important and regular contact)	2823	+	Good
Vicente & Guadalupe, 2022 [107]	GDS-15	Total Network Size	612	0	Poor
*Longitudinal studies*					
Bisschop et al., 2004 [108]	CES-D (20)	Total Network Size (people in important and frequent contact, except partner)	2278	0	Good
Bui, 2020 [19]	CES-D (11)	Total Network Size Confidant size/network	2200	0 0	Good
Chao, 2011 [109]	CES-D (10)	Number of children/Children network Relatives/Kin size Friendship Size (Contacted at least once a week)	4049	+ + +	Good
Coleman et al., 2022 [110]	GDS-5	Overall network size (number of people in network) Effective size (number of non-overlapping groups with which a person interacts)	113	0 0	Good
Hajek & König, 2016 [111]	CES-D (15)	Number of important people regular in contact	2201	0	Good
Harlow et al., 1991 [112]	CES-D (20)	Total Network Size Family Size Friendship size/network Confident Size (Number of friends and family members outside of the household with whom the respondent had been in touch during the 6 months before interview and total size of the network which additionally included family and friends who lived with the respondent)	545	+ 0 + +	Fair
Holwerda et al., 2023 [113]	CES-D (10)	Number of network members (≥ 18 years) with whom respondent had important/frequent contact	899	0	Good
Kuchibhatla et al., 2012 [78]	CES-D (20)	Total Network Size (summarizing seven variables on number of relatives and close friends)	3973	+	Good
Oxman et al., 1992 [114]	CES-D (20)	Number of close relatives phoning/writing yearly Number of close friends phoning/writing yearly Relatives/Kin size Number of children/Children seen weekly	1962	0 0 0 +	Poor
Reynolds et al., 2020 [38]	CES-D	Number of important people regular in contact	3005	0	Good
Santini et al., 2021 [115]	Euro-D	Total Network Size (number of close relations in the social network; up to 7 persons)	38,300	+	Fair
Schwartz & Litwin, 2017 [116]	Euro-D	Total Network Size (up to 7 persons with whom they discuss important matters)	14,101	0	Good
Stringa et al., 2020 [117]	CES-D	Total Network Size (people in important and regular contact)	2279	+	Fair
Tang et al., 2023 [118]	PHQ-9	Total number of network members with whom respondent could discuss important things	1970	0	Good
Werneck et al., 2023 [119]	Euro-D	Network size (number of people in network)	10,569	+	Good

^a^*n*: Sample size, baseline sample was used in longitudinal studies

^b^Results: 0 indicates no sig. relationship (*p* ≥ 0.05), + indicates sig. relationship (*p* < 0.05)

Depression measures: *BDI* Beck Depression Inventory, *CES-D* Centre of Epidemiologic Studies Depression Scale, *CIDI* Composite International Diagnostic Interview, *EURO-D* EURO geriatric depression scale, *GDS* Geriatric Depression Scale, *KDSKA* Kim Depression Scale for Korean Americans, *MADRS* Montgomery–Åsberg Depression Rating Scale, *PHQ-9* Patient Health Questionnaire

There seems to be no consensus regarding the association of the size of different social spheres and depression outcomes among older adults. While Palinkas et al. [64] and Harada et al. [96] found friend network size to be more important than relative network size, Lee and Chou [98] found these variables to be equally important. Furthermore, Minicuci et al. [103] and Oxman et al. [114] found them equally unimportant for depression outcomes.

There also seems to be no consensus regarding gender differences in the association of network size and depression. While two scholars found a significant association of network size and depression only in women but not men [83, 111], three scholars found no evidence for gender differences [91, 104, 106]. Minicuci et al. [103] found the numbers of relatives with close contacts to only be significantly associated with depression in women but not men, while the number of close contacts was significantly associated with depression in men and women.

#### Network composition

Network composition was primarily measured by forming network typologies through clustering (see Table 4). This method makes it particularly challenging to compare results; however, studies consistently showed that diverse social networks protect against depression compared to more restricted networks [120–129]. Concerning network transitions, individuals remaining in and changing to restricted networks showed significantly higher levels of depression than those remaining in non-restricted networks [130, 131]. Consistently, Sicotte et al. [132] found that an increasing diversity of links (measured by diversity of relationship ties) was associated with lower odds of depressive symptoms. Other studies found no significant association [105, 110]. When prestige occupation scores were used as a diversity measure, higher diversity was associated with lower levels of depression compared to less diverse networks [133]. Conversely, Becker et al. [83] found diverse networks to be less associated with a lack of depressive symptoms compared to those relying solely on their partner as their social network.

**Table 4:** Overview of results: network composition and depression

Author	Depression measure	Social network measure	*N* ^a^	Results ^b^	Quality
*Cross-sectional studies*					
Antonucci et al., 1997 [82]	CES-D	Network composition (all family, mostly family, equal members of family and friends, mostly friends, all friends)	3777	+	Good
Becker et al., 2019 [83]	Euro-D	Network types (partner, children, other relatives, family, friends, diverse)	52,513	+	Poor
Cao et al., 2015 [133]	GDS-30	Network types (prestige occupation scores: low, middle and high network)	928	+	Good
Chi & Chou, 2001 [87]	CES-D (20)	Network composition Of relatives and friends felt close to Of relatives and friends seen once a month (all family, mostly family, equal members of family and friends, mostly friends, all friends)	1106	0 +	Good
Choi & Jeon, 2021 [120]	GDS-15	Network types (men: diverse, restricted couple-focused, restricted-unmarried, social-activity-focused, family focused; women: diverse-married, family-focused, restricted-couple-focused, restricted-unmarried, diverse-unmarried)	4608	+	Good
Fiori et al., 2006 [121]	CES-D (11)	Network types (nonfamily restricted, nonfriends, family, diverse, friends)	1669	+	Good
Golden et al., 2009 [134]	GMS	Network types (locally integrated social network vs. any other sort of network)	1299	+	Good
Gumà & Fernández-Carro, 2021 [135]	Euro-D	Network types (partner and others, only relatives, only friends, mixed composition)	6820	0	Good
Harasemiw et al., 2019 [122]	CES-D (10)	Network types (diverse, family-focused, few children, few friends, restricted)	8782	+	Good
Kim & Lee, 2019 [123]	GDS-15	Network types based on LSNS (Friend, Family, Restricted, Diverse)	1000	+	Fair
Li et al., 2019 [100]	PHQ-9	Proportion kin Proportion female Proportion coresident	3157	0 0 +	Fair
Litwin, 2011 [124]	CES-D (8)	Network types (Diverse, friend, congregant, family, restricted)	1350	+	Fair
Litwin, 2012 [125]	CES-D (8)	Network types (only focusing on family and restricted) Family network Restricted network	1275	0 +	Fair
Mechakra-Tahiri et al., 2010 [136]	ESA-Q	Role diversity: number of different types of relationships that participants had, including those with a partner, adult children, siblings, friends, and members of a community group (low, medium, high)	2670	0	Good
Park et al., 2014 [126]	CES-D (10)	Network types (restricted, couple-focused, friend, diverse)	4251	+	Fair
Park et al., 2018 [127]	GDS-15	Network types (diverse/family, diverse/friend, friend-focused, distant, restricted)	6900	+	Good
Pilehvari et al., 2023 [105]	CES-D (20)	Diversity: Index of Qualitative Variation based on various relationship ties	1170	0	Good
Sicotte et al., 2008 [132]	GDS-15	Diversity: number of different types of relationships each participant had: spouse, children, siblings, relatives/friends (range 0–4)	1714	+	Good
Sohn et al., 2017 [128]	CES-D (20)	Network types (restricted, diverse, congregant-restricted, congregant, family)	795	+	Good
Stoeckel & Litwin, 2016 [137]	Euro-D	Network types (distal children, proximal family, spouse, other family, friend, other, no network)	26,401	+	Fair
Vicente & Guadalupe, 2022 [107]	GDS-15	Proportion of each of the following relational categories: Family Friends Neighbors Workplace Institutional relations	612	0 0 0 0 +	Poor
Webster et al., 2015 [138]	CES-D (11)	Type proportions (geographically distant male youth, geographically close/emotionally distant family, close family)	195	0	Fair
Ye & Zhang, 2019 [129]	GDS-15	Network types (diverse, restricted, family-restricted, family, friends)	405	+	Fair
*Longitudinal studies*					
Bui, 2020 [19]	CES-D (11)	Proportion female	2200	0	Good
Chao, 2011 [109]	CES-D (10)	Proportion of close family members (spouses, children, and grandchildren) in the network	4049	+	Good
Coleman et al., 2022 [110]	GDS-15	Proportion of alters in the network with whom ego has a very close relationship Proportion of alters in the network with whom ego is in frequent contact Proportion of alters in the network who are related to ego Diversity: number of unique relationship types in a person’s network divided by network size	113	0 0 0 0	Good
Förster et al., 2018 [131]	CES-D (20)	Changes in network types (family dependent, local self-contained, private restricted, restricted mixed)	783	+	Good
Kim et al., 2016 [130]	CES-D (10)	Changes in network types (restricted, modern-family, friend, diverse)	3501	+	Good
Litwin & Levinsky, 2021 [139]	Euro-D	Changes in network types (remains without network, transitions to close-family networks, transition to other networks, transitions from close-family networks, transitions from other networks)	834	+	Fair
Litwin et al., 2020 [140]	Euro-D	Changes in network types (remains in close-family type, remaining in other network types, transition to other network types, transitions to close-family network types)	13,767	+	Fair

^a^*n*: Sample size, baseline sample was used in longitudinal studies

^b^Results: 0 indicates no sig. relationship (*p* ≥ 0.05), + indicates sig. relationship (*p* < 0.05)

Depression measures: *CES-D* Centre of Epidemiologic Studies Depression Scale, *EURO-D* EURO geriatric depression scale, *ESA-Q* Enquête sur la Santé des Aînés Questionnaire, *GDS* Geriatric Depression Scale, *GMS* Geriatric Mental State, *PHQ-9* Patient Health Questionnaire

Some studies included the share of particular social aspects, such as gender, family, or friends. Consistently, the proportions of females or kin were not identified as significant predictors of depression [19, 100, 107, 138]. Furthermore, there was no consensus about the composition of family and friends. Social networks primarily consisting of family were found to buffer depression more than networks primarily consisting of friends [82, 87]. This was also the case for network transitions [140]. Conversely, Fiori et al. [121] found that the absence of family within a friend context was less detrimental than the absence of friends within a family context. Also, Chao [109] identified that a network proportion of 25–50% family and 50–75% friends was the most advantageous for preventing depression.

While two scholars found no evidence for gender differences in the association of network composition and depression in older adults [132, 136], Choi and Jeon [120] identified gender-specific network types and their association with depression to differ by gender. They found that restricted social network types were associated with increased depressive symptoms in both men and women, whereas a family-centered network was associated with more depressive symptoms only in women.

#### Contact frequency

Less consistency was found in social interaction frequency’s influence on depression in older adults (see Table 5). The cross-sectional studies found 14 significant and 15 insignificant associations. In contrast, among the longitudinal studies, only one significant piece of evidence was found [109], while six effects were identified as insignificant. Three effects were found to be significant only in certain population groups [141, 142]. Furthermore, Blumstein et al. [35] found a significant negative association between weekly contact with friends and children and depression cross-sectionally; this became insignificant when examined longitudinally. Although cross-sectional results are inconclusive, this could indicate that the frequency of contact has the potential to buffer depression at the time of the event but is not necessarily a sustainable buffer for depression.

**Table 5:** Overview of results: contact frequency and depression

Author	Depression measure	Social network measure	*N* ^a^	Results ^b^	Quality
*Cross-sectional studies*					
Becker et al., 2019 [83]	Euro-D	Contact index: contact with each person in network over the last 12 months (daily, several times a week, about once a week, about every two weeks, about once a month, less than once a month, never)	52,513	+	Poor
Blumstein et al., 2004 [35]	CES-D (20)	Weekly contact with friends Weekly contact with children	1290	+ +	Poor
Castro-Costa et al., 2008 [143]	GHQ-12	Weekly frequency of visits from offspring, relatives and friends	1510	0	Poor
Chi & Chou, 2001 [87]	CES-D (20)	Contact frequency with relatives Contact frequency with friends (Less than once a month, once a month, two to three times a month, once a week, two to six times a week, everyday)	1106	+ 0	Good
Domènech-Abella et al., 2017 [89]	CIDI 3.0	Contact with network members at least once per month in the previous 12 months	3535	0	Good
Ermer & Proulx, 2022 [91]	CES-D (11)	Contact with network member (every day, several times a week, once a week, once every two weeks, once a month, a couple times a year, once a year, and less than once a year)	865	0	Fair
Forsman et al., 2012 [144]	GDS-4	Contact frequency with friends Contact frequency with neighbors (Frequent contact: several times a week, several times a month; infrequent contact: few times a year, never, does not exist)	6838	+ +	Good
Jeon & Lubben, 2016 [97]	CES-D (20)	Contact frequency with non-kin Contact frequency with kin (Less than once a month, monthly, 2–3 times a month, weekly, 2–3 times a week, daily)	424	0 +	Fair
La Gory & Fitzpatrick, 1992 [145]	CES-D (20)	Contact scale: visiting friends and relatives, being visited by them, phoning or writing them and meeting them in a social setting	725	+	Poor
Lee et al., 1996 [99]	CES-D (20)	Contact frequency with children Contact frequency with friends (Monthly or less, almost weekly, almost daily)	162	+ +	Poor
Li et al., 2019 [100]	PHQ-9	Average contact frequencies that a participant talked to network members in the past one year (less than once a year to every day)	3157	0	Fair
Litwin & Levinsky, 2022 [146]	Euro-D	In-person contact Electronic contact (daily, several times a week, about once a week, less often, never)	33,403	+ 0	Good
Litwin & Levinsky, 2023 [101]	Euro-D	Contact to confidants (7-point scale: 1 = never; 7 = daily)	35,145	+	Good
Litwin et al., 2015 [102]	Euro-D	Contact frequency (never to daily) to network persons	25,245	0	Good
Marshall & Rue, 2012 [147]	CES-D (20)	Index of contact frequency to family members/ friends/ church members (never to nearly every day)	1108	+	Good
Marshall-Fabien & Miller, 2016 [148]	CES-D (12)	Index of contact frequency to family members/ friends/ church members (never to nearly every day)	1108	0	Good
Minicuci et al., 2002 [103]	CES-D (20)	Personal contact with family members Telephone contact with family members (never, every 6 months, every 2–3 months, every month, more often)	2398	0 0	Good
Palinkas et al., 1990 [64]	BDI (18)	Frequency of face-to-face contact with close family and friends (at least once a week vs. less than once a week)	1615	0	Poor
Pilehvari et al., 2023 [105]	CES-D (20)	Contact to people that immediately surround them (0 = have never spoken to each other to 8 = every day)	1170	0	Good
Taylor et al., 2018 [149]	CES-D (12)	Contact frequency with family members and friends (no isolation: nearly every day, at least once a week, a few times a month; isolation: at least once a month, a few times a year, hardly ever or never) to combination variable (objectively isolated from both family members and friends, objectively isolated from family only, objectively isolated from friends only, not objectively isolated from family and friends)	1439	0	Good
Vicente & Guadalupe, 2022 [107]	GDS-15	Contact frequency (1 = a few times per year to 5 = daily)	612	0	Poor
Wu et al., 2017 [150]	CES-D (20)	Interpersonal contacts over the past year (dichotomized: poor social support was defined as ≤ 1 episode of contact with neighbors, relatives, or friends per month)	5635	+	Good
*Longitudinal studies*					
Blumstein et al., 2004 [35]	CES-D (20)	Weekly contact with friends Weekly contact with children	746	0 0	Good
Bui, 2020 [19]	CES-D (11)	Contact frequency with named alters (less than once a year to every day)	2200	0	Good
Chao, 2011 [109]	CES-D (10)	Contact frequency (mean frequency of meeting with children who were not living with respondent; never or not available to everyday)	4049	+	Good
Gan & Best, 2021 [141]	CES-D (8)	In-person contact with friends Tele-conversation with friends Contact with neighbors (Less than once a month to three or more times a week)	3105	0 0 0/ + (+ only in average outcome profile)	Fair
Husaini, 1997 [142]	CES-D (20)	Contact frequency with friends Contact frequency with relatives (Daily to once a year)	1200	0/ + 0/ +	Poor
Schwartz & Litwin, 2017 [116]	Euro-D	Contact frequency to alters (daily to never)	14,101	0	Good

^a^*n*: Sample size, baseline sample was used in longitudinal studies

^b^Results: 0 indicates no sig. relationship (*p* ≥ 0.05), + indicates sig. relationship (*p* < 0.05)

Depression measures: *BDI* Beck Depression Inventory, *CES-D* Centre of Epidemiologic Studies Depression Scale, *CIDI* Composite International Diagnostic Interview, *EURO-D* EURO geriatric depression scale, *GDS* Geriatric Depression Scale, *GHQ* General Health Questionnaire, *MADRS* Montgomery–Åsberg Depression Rating Scale, *PHQ-9* Patient Health Questionnaire

There was no consensus among studies about the association of depression with contact frequencies in particular social spheres, such as friends, children, and non-kin [35, 64, 87, 97, 99, 109, 141–145, 149]. Chi and Chou [87] found contact frequency with relatives to be more advantageous in buffering depression than the frequency of contact with friends. In contrast, Jeon and Lubben [97] found contact frequency with non-kin to be negatively associated with depressive symptoms in older Korean immigrants, while contact frequency with kin was not significantly associated.

Only two scholars accounted for gender differences in the association of contact frequency and depression among older adults. Ermer and Proulx [91] found no significant association of contact frequency and depression in women or men. In their cross-sectional analysis, Blumstein et al. [35] also found no gender differences in the association between weekly contact with children and depression, but identified weekly contact with friends to only be significantly associated with depression in women but not men. However, these gender differences did not hold longitudinally.

#### Density

Four articles examined how social network density was associated with depression in older adults (see Table 6). The results were inconclusive, cross-sectionally as well as longitudinally. Coleman et al. [110] and Vicente and Guadalupe [107] found no significant associations. Furthermore, the significant associations found were contradictory even though the same data and measurements were used. Dorrance Hall et al. [90] found that confidant network density was negatively associated with levels of depression cross-sectionally. In contrast, Bui [19] conducted a longitudinal study and found that a higher network density was significantly associated with increased depressive symptoms.

**Table 6:** Overview of results: network density and depression

Author	Depression measure	Social network measure	*N* ^a^	Results ^b^	Quality
*Cross-sectional studies*					
Dorrance Hall et al., 2019 [90]	CES-D (9)	Number of observed links divided by perceived potential links among network members (indicated by respondent; links is being defined as speaking on a monthly basis)	2249	+	Good
Vicente & Guadalupe, 2022 [107]	GDS-15	Proportion of network members that knows one another; calculated by dividing the number of actual connections between network members by the number of potential connections	612	0	Poor
*Longitudinal studies*					
Bui, 2020 [19]	CES-D (11)	Ratio of actual ties to perceived possible ties (indicated by respondent; ties is being defined as having any contact)	2200	+	Good
Coleman et al., 2022 [110]	GDS-5	Mean of closeness of the tie between alters	113	0	Good

^a^*n*: Sample size, baseline sample was used in longitudinal studies

^b^Results: 0 indicates no sig. relationship (*p* ≥ 0.05), + indicates sig. relationship (*p* < 0.05)

Depression measures: *CES-D* Centre of Epidemiologic Studies Depression Scale; *GDS* Geriatric Depression Scale

#### Geographic proximity

Three cross-sectional articles considered geographical proximity as a social network determinant for depression among older adults (see Table 7). No study focused on the respective relationship longitudinally. All the articles found significant but inconclusive results. While Litwin et al. [102] and Vicente and Guadalupe [107] found that geographically closer social networks buffer depression, Becker et al. [83] identified that geographically closer social networks increased depression. This may be attributable to the measurement used to assess geographic proximity: Litwin et al. [102] included individuals living within the respondent’s household, while Becker et al. [83] did not. This strongly suggests that the direction of effects is dependent on operationalization.

**Table 7:** Overview of results: geographic proximity and depression

Author	Depression measure	Social network measure	*N* ^a^	Results ^b^	Quality
*Cross-sectional studies*					
Becker et al., 2019 [83]	Euro-D	Proximity index (Average geographical proximities to network members: more than 500 km, 100 to 500 km, 25 to 100 km, 5 to 25 km, 1 to 5 km, and less than 1 km)	52,513	+	Poor
Litwin et al., 2015 [102]	Euro-D	Proximity (Scores ranged from “more than 500 km away” (1) to “in the same household” (8))	25,245	+	Good
Vicente & Guadalupe, 2022 [107]	GDS-15	Proximity index (Average of geographical proximities to network members; more than 50 km, less than 50 km, in the same city/village, in the same street/neighborhood, in the same household)	612	+	Poor

^a^*n*: Sample size, baseline sample was used in longitudinal studies

^b^Results: 0 indicates no sig. relationship (*p* ≥ 0.05), + indicates sig. relationship (*p* < 0.05)

Depression measures: *EURO-D* EURO geriatric depression scale; *GDS* Geriatric Depression Scale

#### Homogeneity

Furthermore, two cross-sectional studies examined homo-/heterogeneity (see Table 8). Their evidence suggested no significant relationship between network homo-/heterogeneity and depression among older adults. Goldberg et al. [94] determined network homogeneity through questions about the sex, age, and religion of all network members. They found no significant association with depression. Murayama et al. [151] measured homo-/heterogeneity through respondents’ perceptions of the (dis)similarity of characteristics. They found a significant negative association with depression. This was only found for individuals with a strongly homogenous network and not for those with a weakly homogenous network. No significant relationship was found between network heterogeneity and depression outcomes.

**Table 8:** Overview of results: network homogeneity and depression

Author	Depression measure	Social network measure	*N* ^a^	Results ^b^	Quality
*Cross-sectional studies*					
Goldberg et al., 1985 [94]	CES-D (20)	Homogeneity determined by questions about sex, age, and religion of all network members	1104	0	Good
Murayama et al., 2015 [151]	GDS-15	Homogeneity Heterogeneity (Perceived (dis)similarity to network members regarding social characteristics age, gender, and SES)	6416	+ 0	Fair

^a^*n*: Sample size, baseline sample was used in longitudinal studies

^b^Results: 0 indicates no sig. relationship (*p* ≥ 0.05), + indicates sig. relationship (*p* < 0.05)

Depression measures: *CES-D* Centre of Epidemiologic Studies Depression Scale, *GDS* Geriatric Depression Scale

### Structural social network variables as outcome variable

Thirteen studies focused on social networks as outcome variables of depression (see Table 9). Seven articles examined this association cross-sectionally, while six articles did so longitudinally.

The articles examining the relationship between depression and social networks specifically focused on social network scale outcomes, network size, network composition, density, and contact frequency.

**Table 9:** Overview of articles focusing on structural network aspects as outcome variable

Author	Depression measure	Social network measure	*N* ^a^	Results ^b^	Quality
*Cross-sectional studies*					
Ali et al., 2022 [152]	NDSM	Composition (large with strain; large without strain; small, diverse, low contact; small, restricted, high contact; medium size and support)	5192	+	Good
Bincy et al., 2022 [153]	GDS-15	Scale (LSNS)	1000	+	Good
Li et al., 2022 [100]	GDS-15	Scale (LSNS)	2267	+	Good
Merchant et al., 2020 [154]	GDS	Scale (LSNS)	202	0	Fair
Shouse et al., 2013 [155]	GDS-15	Network size (Hierarchical mapping technique) Total Inner circle Middle circle Outer circle	79	+ + + 0	Fair
Sugie et al., 2022 [37]	GDS-15	LSNS (dichotomous, scores < 12 limited network)	268	+	Good
Wendel et al., 2022 [156]	GDS	Scale (LSNS) Total Family subscale Friends subscale	1030	+ + +	Good
*Longitudinal studies*					
Bui, 2020 [19]	CES-D (11)	Network size: Total network size Number of close ties Composition: Proportion female Density: ratio of actual ties to theoretically possible ties Contact frequency (less than once a year to every day)	2200	0 + 0 0 0	Good
Domènech-Abella et al., 2019 [20]	CIDI-SF	Scale (SNI)	5066	0	Good
Houtjes et al., 2014 [157]	CES-D (20)	Network size (Socially active relationships of the respondent)	277	+	Good
Reynolds et al., 2020 [38]	CES-D	Network size (Number of important people regular in contact)	3005	0	Good
Voils et al., 2007 [158]	MADRS	Network size (assessed by 4 items, no further specification) Contact frequency (Weekly contact assessed by four items; not at all, once, twice, three times, four times, five times, six times, seven times or more)	339	+ 0	Fair
Zhang et al., 2023 [36]	DASS-21 (depression subscale)	Scale (LSNS)	634	+	Good

^a^*n*: Sample size, baseline sample was used in longitudinal studies

^b^Results: 0 indicates no sig. relationship (*p* ≥ 0.05), + indicates sig. relationship (*p* < 0.05)

Depression measures: *CES-D* Centre of Epidemiologic Studies Depression Scale, *CIDI-SF* Composite International Diagnostic Interview (Short Form), *DASS-21* Depression Anxiety Stress Scale, *GDS* Geriatric Depression Scale, *NDSM* NSHAP Depressive Symptoms Measure

Social network measures: *LSNS* Lubben Social Network Scale, *SNI* Social Network Index

#### Network scales

Evidence about the relationship between depression and network scales was mixed. While Merchant et al. [154] found no evidence cross-sectionally, other scholars found significant evidence that depression was associated with lower scores on network scales [37, 153, 159] and subscales [156]. However, the longitudinal evidence found was contradictory [20, 36].

#### Network size

Depression was primarily identified as a significant predictor for network size. This was found cross-sectionally [155] and longitudinally [19, 157, 158]. Shouse et al. [155] found depression to be a predictor for a smaller inner circle network size. Furthermore, Bui [19] found that depressive symptoms significantly affected an individual’s number of close ties but not total social network size. In contrast, Houtjes et al. [157] examined differences in network size depending on depression course types. They found decreasing network sizes for all depression course types in older adults.

#### Network composition

Cross-sectionally, Ali et al. [152] found that individuals with more depressive symptoms had smaller and more strained networks. Bui [19] did not identify depressive symptoms as a significant predictor of the proportion of females in an individual’s network.

#### Contact frequency

No significant evidence suggested that depression affects contact frequency [19, 158].

#### Network density

Bui [19] did not find depressive symptoms to significantly predict network density.

### Reciprocal relationship of structural network aspects and depression

Only five articles examined the relationship between structural network aspects and depression reciprocally [19, 20, 36–38]. However, no reciprocal relationship was found between depression and network size [19, 38], composition [19], contact frequency [19], and network scales [20, 36, 37]. Bui [19] only identified greater network density to significantly reduce depressive symptoms 5 years later, but not the other way around. Network size, number of close ties, contact frequency, or network composition did not significantly affect depressive symptoms 5 years later. Furthermore, Domènech-Abella et al. [20] found that the social network index significantly affects depression longitudinally; however, this relationship was not reciprocal. In contrast, Zhang et al. [36] found that higher depression scores at baseline predicted lower social network scores at a 6-month follow-up. However, social network scores did not predict depression at a 6-month follow-up. Bui [19] found more depressive symptoms to be associated with fewer close ties 5 years later. However, all other structural network measures (network size, composition, and contact frequency) were insignificant; therefore, the author concluded that there was no clear reciprocal relationship between structural network measures and depression [19].

### Importance of functional network aspects

Thirty articles included social support in their analysis and examined whether social networks’ structural or functional aspects were more important in predicting depression outcomes in older adults. Singh et al.’s [44] article was excluded because social support measures’ effect sizes and significance were not presented.

However, no consensus can be reached. Seven studies identified structural aspects as more critical in predicting depression in terms of significant effects [35, 53, 54, 74, 98, 106, 117], while nine scholars found social support to be more relevant [34, 62, 82, 95, 107, 108, 110, 114, 129]. Sixteen studies found that social support and social network aspects were equally (not) predictive of depressive symptoms [19, 80, 85–87, 90, 92, 103, 109, 118, 122, 132, 133, 136, 138, 142].

## Discussion

### Social network characteristics and depression among older adults

This study aimed to systematize the evidence about the relationship between social networks and depression in older adults. It focused on the structural aspects of social networks because these are particularly suited for understanding their association with critical health outcomes [14–16]. It differentiated between the causality of relationships and structural and functional social network characteristics’ impact on depression.

Most articles followed the main-effect model [17] and considered depression as an outcome variable of social network characteristics in examining the relationship between structural social network aspects and depression among older adults. Only eight articles exclusively accounted for the reversed logic of causality: social network characteristics as an outcome of depression [152–159]. Five out of 127 articles examined the reciprocal relationship between structural social network characteristics and depression [19, 20, 36–38]. However, these articles found no clear reciprocal relationship. Therefore, no theoretical conclusions can be drawn based on these findings.

The majority of articles focused on depression as an outcome of older adults’ social network characteristics. They primarily used cross-sectional evidence. Structural network characteristics were predominantly operationalized through network scales, size, composition, and contact frequency. Conversely, they generally neglected network density, homogeneity, and geographical proximity. Evidence about whether and how the latter three social network aspects affect depression outcomes in older adults was inconsistent [19, 83, 90, 94, 102, 107, 110, 151]. Most evidence supported the assumption that higher scores on social network scales buffer depression [20, 37, 41–43, 45–54, 56–58, 61, 63–66, 68–72, 74–76, 79–81]. Corroborating previous literature reviews [2, 13], some evidence suggested that a more extensive network size buffers depression outcomes in older adults compared to a smaller network size [33, 64, 78, 82, 83, 85–87, 90, 92–94, 96, 98–102, 106, 109, 112, 114, 115, 117, 119]. Three quarters of the studies also identified that network composition was significantly associated with depression outcomes in older adults; diverse social networks were found to be more beneficial than restricted networks [120–131]. This aligns with Santini et al.’s [13] findings, who consistently identified diverse types of social networks as associated with favorable depression outcomes. Results on the effect of contact frequency on depression were less consistent: no clear evidence was found cross-sectionally, and no substantial effects of contact frequency were found in longitudinal studies. This confirms Schwarzbach et al.’s [3] findings, which reported inconsistent results cross-sectionally and longitudinally.

Furthermore, the effects of social network aspects on depression seem to be largely stable for women and men [35, 43, 47, 50, 60, 66, 68, 80, 83, 91, 103, 104, 106, 111, 120, 132, 136, 151]. Notably, no consensus can be reached about whether family or friends are more critical for favorable depression outcomes in older adults [41–44, 82, 87, 109, 121, 140]. This challenges the previous assumption that family is the most crucial source of good health [160].

A minority of articles found social network characteristics to be outcomes of depression. While depression did not influence density [19] and contact frequency [19, 158], an unclear effect was found for network scales [20, 36, 37, 153, 154, 156, 159] and network composition [19, 152]. However, depression significantly reduced the size of an individual’s social network and their number of close relationships [19, 155, 157, 158].

This review does not confirm the previous systematic reviews’ findings [3, 13] that social networks’ functional aspects are more important than their structural aspects in predicting depression. The articles that considered functional network characteristics showed no consensus about whether structural or functional network aspects were more important in buffering depression outcomes in older adults [19, 34, 35, 53, 54, 62, 74, 80, 82, 85–87, 90, 92, 95, 98, 103, 106–110, 114, 117, 118, 122, 129, 132, 133, 136, 138, 142].

Furthermore, very few studies reported effect sizes. However, the studies that reported standardized coefficients almost exclusively identified small effect sizes across all structural social network aspects [41, 43, 47, 51–56, 58, 59, 61, 63–66, 85–87, 93, 96, 99, 101, 102, 104, 112, 120, 121, 123, 125, 126, 128, 129, 133, 137, 139, 140, 147, 153, 159]. Although the studies covered a wide sample size range, there were no differences in the results. This suggests that structural network aspects have a rather small but stable influence on depression. However, future studies should report effect sizes (e.g., by standardized coefficients) to ensure the comparability of studies and individual effects.

### Limitations and future implications

This systematic review is the first to specifically focus on the relationship between structural social network aspects and depression outcomes among older adults. While previous systematic reviews have been helpful, they have loosely applied the constructs of social networks and limited their focus to particular geographic areas. Additionally, the vast body of evidence that has emerged during the last decade highlights the importance of this updated systematic review. However, our review has some limitations. Like other reviews, the articles included in this review may be prone to publication bias. In addition, we did not use controlled vocabulary terms such as MeSH and Psychological Index Terms in our search strategy. As our search strategy and keywords were informed by other reviews [2, 3, 5–8, 13, 23–25], we used a diverse range of keywords relevant to the field. Our comprehensive search strategy is reflected in the high number of initial articles found. Consequently, we anticipate having identified all relevant articles. Furthermore, we only included articles published in English, neglecting the findings reported in different languages. However, we did this to counteract possible regional bias induced by language knowledge of the authors. Additionally, the exclusion of non-English articles was found to have minimal impact on the results and overall conclusions of a review [161, 162]. However, future research could employ machine translation to counteract selection bias induced by language restrictions. This should be particularly beneficial in contexts in which limited evidence exists.

Further, it must be emphasized that we focused on community-dwelling older adults, excluding institutionalized individuals from analysis. It should be acknowledged that regional bias may arise, given the different proportions of older adults living in institutions across countries. However, we decided to do this as institutionalized individuals are likely to have predetermined social networks which may affect depression outcomes differently.

Additionally, the use of the term “social network” may exclude studies focusing solely on family networks, which are highly relevant for the mental health of older adults. However, as the individual network should not be limited to family networks alone, we have deliberately opted for the holistic term here, to capture the social network in its entirety. This approach is supported by the ambiguous results on the importance of family and friendship relationships for depression among older adults (see analysis above).

Furthermore, this systematic review included studies from peer-reviewed journals, excluding gray literature. This may limit our findings. However, it ensures that the included articles are high quality. Furthermore, systematic reviews do not allow qualitative studies to be included. While qualitative studies are limited in their potential to establish causal relationships between variables, they provide valuable insights into the understanding and interpretation of psychosocial phenomena that quantitative research often cannot access.

This systematic review aimed to understand the potential of structural social network characteristics holistically by reviewing them all and not limiting the focus on only a few. That is why we did not conduct a meta-analysis. Firstly, evidence is too small to be statistically analyzed, such as in the social network domains network density, homogeneity, and geographical proximity. Secondly, particularly in the social network domain composition, results are not necessarily comparable since cluster analysis results in different numbers of clusters which are consequently characterized differently. However, future research should conduct a meta-analysis with the more comparable domains network scale, size, and contact frequency.

Despite this review’s limitations, its strength lies in its systematic search; multiple keywords and broad terminologies were used to capture as many articles as possible. This is reflected in the significant number of publications included in this review.

Much of the evidence reported here came from cross-sectional studies. Additionally, only eight of the 127 articles exclusively considered social networks as dependent variables, and only five studies examined the reciprocal relationship. This makes it particularly difficult to draw causal conclusions about the relationship between social networks and depression among older adults. Further research is needed to disentangle the reciprocal relationship using longitudinal data. Furthermore, limited literature focused on the relationship between depression and network density, homogeneity, and geographical proximity. Additionally, these results were inconclusive. Therefore, these relationships should be closely examined in future research.

## Conclusion

This review gathered evidence and confirmed that having larger and more diverse social networks and closer ties buffers depression among older adults. Evidence about the relationship between contact frequency and depression was inconclusive. Literature on the relationships between depression and network density, homogeneity, and geographical proximity is scarce and inconclusive; therefore, further research is needed. Although this review examined a vast body of research about the relationship between social network aspects and depression among older adults, no conclusions about causality could be drawn. Contrary to other reviews, the evidence suggests that functional and structural networks are equally important in determining depression outcomes in older adults.

This review highlights that quantifying older adults’ social relations is crucial to understanding depression outcomes in older adults. As the population ages and multimorbidity and social isolation increase, appropriate social gerontological interventions are needed. Based on this review, interventions could potentially promote the integration of older adults into larger and more diverse social settings. Following the recommendations of a systematic review about the effectiveness of interventions targeting social isolation in older adults [163], group interventions like social activities are the most effective in broadening older adults’ social networks and increasing their contacts. These interventions can help to counteract depression in older adults.

### Supplementary Information

Supplementary Material 1.

## Data Availability

The datasets used and/or analyzed during the current study are available from the corresponding author on reasonable request. All data generated or analyzed during this study are included in this published article.
