# Supplementary material for: The association of social networks and depression in community-dwelling older adults: a systematic review
Source: Syst Rev. 2024 Jun 20;13:161. doi: 10.1186/s13643-024-02581-6 (PMC11188217; doi:10.1186/s13643-024-02581-6)
Supplement: Supplementary file 1 — Supplementary Material 1. [file 13643_2024_2581_MOESM1_ESM.docx]

Table A1 PRISMA Checklist

| Topic | No. | Item | Location where item is reported |
| --- | --- | --- | --- |
| TITLE |  |  |  |
| Title | 1 | Identify the report as a systematic review. | Title |
| ABSTRACT |  |  |  |
| Abstract | 2 | See the PRISMA 2020 for Abstracts checklist | Abstract |
| INTRODUCTION |  |  |  |
| Rationale | 3 | Describe the rationale for the review in the context of existing knowledge. | Introduction |
| Objectives | 4 | Provide an explicit statement of the objective(s) or question(s) the review addresses. | Introduction |
| METHODS |  |  |  |
| Eligibility criteria | 5 | Specify the inclusion and exclusion criteria for the review and how studies were grouped for the syntheses. | 2.1 Eligibility criteria |
| Information sources | 6 | Specify all databases, registers, websites, organisations, reference lists and other sources searched or consulted to identify studies. Specify the date when each source was last searched or consulted. | 2.2 Search strategy |
| Search strategy | 7 | Present the full search strategies for all databases, registers and websites, including any filters and limits used. | 2.2 Search strategy; Review-protocol online: <https://doi.org/10.17605/OSF.IO/6QDPK> |
| Selection process | 8 | Specify the methods used to decide whether a study met the inclusion criteria of the review, including how many reviewers screened each record and each report retrieved, whether they worked independently, and if applicable, details of automation tools used in the process. | 2.3 Study selection |
| Data collection process | 9 | Specify the methods used to collect data from reports, including how many reviewers collected data from each report, whether they worked independently, any processes for obtaining or confirming data from study investigators, and if applicable, details of automation tools used in the process. | 2.4 Data extraction |
| Data items | 10a | List and define all outcomes for which data were sought. Specify whether all results that were compatible with each outcome domain in each study were sought (e.g. for all measures, time points, analyses), and if not, the methods used to decide which results to collect. | 2.4 Data extraction |
|  | 10b | List and define all other variables for which data were sought (e.g. participant and intervention characteristics, funding sources). Describe any assumptions made about any missing or unclear information. | 2.4 Data extraction |
| Study risk of bias assessment | 11 | Specify the methods used to assess risk of bias in the included studies, including details of the tool(s) used, how many reviewers assessed each study and whether they worked independently, and if applicable, details of automation tools used in the process. | 2.5 Quality appraisal |
| Effect measures | 12 | Specify for each outcome the effect measure(s) (e.g. risk ratio, mean difference) used in the synthesis or presentation of results. | n.a. |
| Synthesis methods | 13a | Describe the processes used to decide which studies were eligible for each synthesis (e.g. tabulating the study intervention characteristics and comparing against the planned groups for each synthesis (item 5)). | 2.6 Synthesis method |
|  | 13b | Describe any methods required to prepare the data for presentation or synthesis, such as handling of missing summary statistics, or data conversions. | n.a. |
|  | 13c | Describe any methods used to tabulate or visually display results of individual studies and syntheses. | 2.6 Synthesis method |
|  | 13d | Describe any methods used to synthesize results and provide a rationale for the choice(s). If meta-analysis was performed, describe the model(s), method(s) to identify the presence and extent of statistical heterogeneity, and software package(s) used. | n.a. |
|  | 13e | Describe any methods used to explore possible causes of heterogeneity among study results (e.g. subgroup analysis, meta-regression). | n.a. |
|  | 13f | Describe any sensitivity analyses conducted to assess robustness of the synthesized results. | n.a. |
| Reporting bias assessment | 14 | Describe any methods used to assess risk of bias due to missing results in a synthesis (arising from reporting biases). | n.a. |
| Certainty assessment | 15 | Describe any methods used to assess certainty (or confidence) in the body of evidence for an outcome. | n.a. |
| RESULTS |  |  |  |
| Study selection | 16a | Describe the results of the search and selection process, from the number of records identified in the search to the number of studies included in the review, ideally using a flow diagram. | Figure 1 |
|  | 16b | Cite studies that might appear to meet the inclusion criteria, but which were excluded, and explain why they were excluded. | Additional file 1, Table A2 |
| Study characteristics | 17 | Cite each included study and present its characteristics. | Table 2 – Table 9 |
| Risk of bias in studies | 18 | Present assessments of risk of bias for each included study. | n.a. |
| Results of individual studies | 19 | For all outcomes, present, for each study: (a) summary statistics for each group (where appropriate) and (b) an effect estimate and its precision (e.g. confidence/credible interval), ideally using structured tables or plots. | Table 2 – Table 9 |
| Results of syntheses | 20a | For each synthesis, briefly summarise the characteristics and risk of bias among contributing studies. | 3 Results |
|  | 20b | Present results of all statistical syntheses conducted. If meta-analysis was done, present for each the summary estimate and its precision (e.g. confidence/credible interval) and measures of statistical heterogeneity. If comparing groups, describe the direction of the effect. | 3 Results |
|  | 20c | Present results of all investigations of possible causes of heterogeneity among study results. | 3 Results |
|  | 20d | Present results of all sensitivity analyses conducted to assess the robustness of the synthesized results. | n.a. |
| Reporting biases | 21 | Present assessments of risk of bias due to missing results (arising from reporting biases) for each synthesis assessed. | n.a. |
| Certainty of evidence | 22 | Present assessments of certainty (or confidence) in the body of evidence for each outcome assessed. | n.a. |
| DISCUSSION |  |  |  |
| Discussion | 23a | Provide a general interpretation of the results in the context of other evidence. | 4.1 Social network characteristics and depression among older adults |
|  | 23b | Discuss any limitations of the evidence included in the review. | 4.1 Social network characteristics and depression among older adults |
|  | 23c | Discuss any limitations of the review processes used. | 4.2 Limitations and future implications |
|  | 23d | Discuss implications of the results for practice, policy, and future research. | 4.2 Limitations and future implications, 5 Conclusion |
| OTHER INFORMATION |  |  |  |
| Registration and protocol | 24a | Provide registration information for the review, including register name and registration number, or state that the review was not registered. | 2 Methods |
|  | 24b | Indicate where the review protocol can be accessed, or state that a protocol was not prepared. | 2 Methods |
|  | 24c | Describe and explain any amendments to information provided at registration or in the protocol. | n.a. |
| Support | 25 | Describe sources of financial or non-financial support for the review, and the role of the funders or sponsors in the review. | Funding |
| Competing interests | 26 | Declare any competing interests of review authors. | Competing interests |
| Availability of data, code and other materials | 27 | Report which of the following are publicly available and where they can be found: template data collection forms; data extracted from included studies; data used for all analyses; analytic code; any other materials used in the review. | up on request |

Table A2 Excluded studies and exclusion reason

| **Authors** | **Title** | **Exclusion reason** |
| --- | --- | --- |
| Adams et al., 2004 | Loneliness and Depression in Independent Living Retirement Communities: Risk and Resilience Factors | Institutionalized population/ no separate analysis for community-dwelling population |
| Adams et al., 2023 | The Risk for Loneliness and Major Depression among Solo Agers | No structural measure of social network |
| Allen et al., 2022 | Longitudinal Cohort Study of Depression and Anxiety Among Older Informal Caregivers Following the Initial COVID-19 Pandemic Response in Aotearoa New Zealand | No structural measure of social network |
| Ang, 2022 | Changing Relationships Between Social Contact, Social Support, and Depressive Symptoms During the COVID-19 Pandemic | No structural measure of social network |
| Baek et al., 2021 | Gender differences in the longitudinal association between husbands' and wives' depressive symptoms among Korean older adults: the moderating effects of the spousal relationship | No structural measure of social network |
| Baiyewu et al., 2015 | Depression in elderly people living in rural Nigeria and its association with perceived health poverty and social network | No structural measure of social network |
| Baker et al., 1996 | Screening African-American elderly for the presence of depressive symptoms: A preliminary investigation | Not adjusted for confounders |
| Barnes et al., 2022 | Cumulative effect of loneliness and social isolation on health outcomes among older adults | No association between depression and social network measured |
| Bartucz et al., 2022 | The Protective Effect of Culture on Depression During Covid-19 Pandemic: A Romanian National Study | No structural measure of social network |
| Bassett & Moore, 2013 | Social capital and depressive symptoms: The association of psychosocial and network dimensions of social capital with depressive symptoms in Montreal Canada | Non-matching age restrictions |
| Beekman et al., 2002 | The impact of depression on the well-being disability and use of services in older adults: A longitudinal perspective | No association between depression and social network measured |
| Bélanger et al., 2016 | Sources of social support associated with health and quality of life: a cross-sectional study among Canadian and Latin American older adults | No structural measure of social network |
| Bianchi et al., 2023 | Structure of personal networks and cognitive abilities: A study on a sample of Italian older adults | No association between depression and social network measured |
| Biegel et al., 1991 | Social support networks of White and Black elderly people at risk for institutionalization | No association between depression and social network measured |
| Bijnsdorp et al., 2018 | Het combineren van meerdere rollen onder ouderen: vermindert of verbetert dit het welbevinden? | Other language |
| Bizzozero-Peroni et al., 2022 | Proinflammatory dietary pattern and depression risk in older adults: Prospective analyses from the Seniors-ENRICA studies | No structural measure of social network |
| Blazer, 1983 | Impact of late-life depression on the social network | No structural measure of social network |
| Boey, 1999 | Cross-validation of a short form of the CES-D in Chinese elderly | Institutionalized population/ no separate analysis for community-dwelling population |
| Bowling & Farquhar, 1991 | Associations with social networks, social support, health status and psychiatric morbidity in three samples of elderly people | No measure of depression |
| Burger et al., 2020 | Bereavement or breakup: Differences in networks of depression | No association between depression and social network measured |
| Buys et al., 2008 | Prevalence and predictors of depressive symptoms among rural older Australians and Americans | No association between depression and social network measured |
| Canbal et al., 2012 | Effects of depression and life factors on social network score in elderly people in Cankaya Ankara | Patient group |
| Cao et al., 2023 | The impact of hearing loss on cognitive impairment: The mediating role of depressive symptoms and the moderating role of social relationships | No association between depression and social network measured |
| Cappeliez et al., 2007 | Recovery from depression in older depressed patients in primary care: Relation with depression severity and social support | Intervention |
| Castell-Alcalá et al., 2022 | Evolution of physical function, cognition, depressive mood, and quality of life during the Covid-19 pandemic in prefrail elderly people: A longitudinal cohort study (Covid-Mefap) | Patient group |
| Cené et al., 2022 | Social Isolation and Incident Heart Failure Hospitalization in Older Women: Women’s Health Initiative Study Findings | No association between depression and social network measured |
| Chang, 2019 | Cross-cultural comparative study of psychological distress between older Korean immigrants in the United States and older Koreans in South Korea | No measure of depression |
| Chen et al., 2016 | Neighborhood support network perceived proximity to community facilities and depressive symptoms among low socioeconomic status Chinese elders | No structural measure of social network |
| Chen et al., 2019 | The influence of social support on loneliness and depression among older elderly people in China: Coping styles as mediators | No structural measure of social network |
| Chen et al., 2022 | Depression and PTSD in the aftermath of strict COVID-19 lockdowns: a cross-sectional and longitudinal network analysis | Non-matching age restrictions |
| Chen et al., 2023 | Later-life depressive symptoms during the Covid-19 pandemic: Investigations of individual, cumulative, and synergistic effects of social isolation | No structural measure of social network |
| Child & Lawton, 2020 | Personal networks and associations with psychological distress among young and older adults | No measure of depression |
| Choi & Lee, 2022 | Factors Affecting Depression in Middle-Aged and Elderly Men Living Alone: A Cross-Sectional Path Analysis Model | No structural measure of social network |
| Copeland et al., 1999 | Community-based case-control study of depression in older people. Cases and sub-cases from the MRC-ALPHA Study | Patient group |
| Cornwell & Waite, 2009 | Social Disconnectedness Perceived Isolation and Health among Older Adults | No structural measure of social network |
| Cui et al., 2022 | The Role of Perceived and Objective Social Connectedness on Risk for Suicidal Thoughts and Behavior in Late-Life and Their Moderating Effect on Cognitive Deficits | No association between depression and social network measured |
| Curran et al., 2019 | Symptom profiles of late-life anxiety and depression: The influence of migration religion and loneliness | No association between depression and social network measured |
| de Feijter et al., 2022 | The network of psychosocial health in middle-aged and older adults during the first covid-19 lockdown | No structural measure of social network |
| De Main et al., 2023 | Longitudinal associations between mental health and social environment in older adults: a multilevel growth modeling | Non-matching age restrictions |
| Dean et al., 1990 | Effects of social support from various sources on depression in elderly persons | No structural measure of social network |
| Djundeva et al., 2019 | Is Living Alone "Aging Alone"? Solitary Living Network Types and Well-Being | No mean age |
| Dobrota et al., 2022 | The association of hearing problems with social network strength and depressive symptoms: the cardiovascular health study | No association between depression and social network measured |
| Domenech-Abella et al., 2021 | Social network size loneliness physical functioning and depressive symptoms among older adults: Examining reciprocal associations in four waves of the Longitudinal Aging Study Amsterdam (LASA) | Not adjusted for confounders |
| Dos Santos et al., 2023 | Positive attributes in elderly people with different degrees of depression: a study based on network analysis | No structural measure of social network |
| Doubova et al., 2010 | Social network types and functional dependency in older adults in Mexico | No association between depression and social network measured |
| DuPertuis et al., 2001 | Does the source of support matter for different health outcomes? Findings from the Normative Aging Study | No structural measure of social network |
| Eymundsdottir et al., 2022 | Social network and the risk for developing mild cognitive impairment and dementia among older adults | No association between depression and social network measured |
| Fernandez et al., 1998 | Moderating the effects of stress on depressive symptoms | No structural measure of social network |
| Field et al., 2002 | Social networks and health of older people living in sheltered housing | Institutionalized population/ no separate analysis for community-dwelling population |
| Finch & Zautra, 1992 | Testing latent longitudinal models of social ties and depression among the elderly: A comparison of distribution-free and maximum likelihood estimates with nonnormal data | No structural measure of social network |
| Fiordelli et al., 2020 | Differentiating objective and subjective dimensions of social isolation and apprasing their relations with physical and mental health in italian older adults | Institutionalized population/ no separate analysis for community-dwelling population |
| Forsell & Winblad, 1999 | Incidence of major depression in a very elderly population | Patient group |
| Freyne et al., 2005 | A longitudinal study of depression in old age I: outcome and relationship to social networks | Patient group |
| Fuhrer et al., 1999 | Psychological disorder and mortality in French older adults: Do social relations modify the association? | No association between depression and social network measured |
| Fuller-Iglesias et al., 2015 | The Complex Nature of Family Support Across the Life Span: Implications for Psychological Well-Being | No structural measure of social network |
| Fuller-Iglesias, 2015 | Social ties and psychological well-being in late life: the mediating role of relationship satisfaction | Institutionalized population/ no separate analysis for community-dwelling population |
| Golden et al., 2009 | Social support network structure in older people: underlying dimensions and association with psychological and physical health | No association between depression and social network measured |
| Gureje et al., 2008 | Determinants of quality of life of elderly Nigerians: results from the Ibadan study of ageing | No association between depression and social network measured |
| Gureje et al., 2011 | Incidence and risk factors for late-life depression in the Ibadan Study of Ageing | Institutionalized population/ no separate analysis for community-dwelling population |
| Győri, 2023 | The impact of social-relationship patterns on worsening mental health among the elderly during the COVID-19 pandemic: Evidence from Hungary | No measure of depression |
| Hajek & König, 2021 | Determinants of psychosocial factors among the oldest old¬†-¬†Evidence from the representative "Survey on quality of¬†life and subjective well-being of the very old in North¬†Rhine-Westphalia (NRW80+)" | Institutionalized population/ no separate analysis for community-dwelling population |
| Hamid et al., 2021 | Do Living Arrangements and Social Network Influence the Mental Health Status of Older Adults in Malaysia? | No association between depression and social network measured |
| Harrison et al., 2010 | Alone? Perceived social support and chronic interpersonal difficulties in suicidal elders | No structural measure of social network |
| Hed et al., 2020 | Gender differences in resources related to depressive symptoms during the early years of retirement: A Swedish population-based study | No structural measure of social network |
| Henderson et al., 1986 | The elderly who live alone: Their mental health and social relationships | No structural measure of social network |
| Herbolsheimer et al., 2018 | Why Is Social Isolation Among Older Adults Associated with Depressive Symptoms? The Mediating Role of Out-of-Home Physical Activity | No structural measure of social network |
| Hill et al., 2023 | Mental health impact of the COVID-19 pandemic in U.S. military veterans: a population-based, prospective cohort study | Non-matching age restrictions |
| Hopper et al., 2023 | Contributors to mental health resilience in middle-aged and older adults: an analysis of the Canadian Longitudinal Study on Aging | No measure of depression |
| Houtjes et al., 2017 | Is the naturalistic course of depression in older people related to received support over time? Results from a longitudinal population-based study | No structural measure of social network |
| Huang et al., 2022 | Hearing loss and depressive symptoms in older Chinese: whether social isolation plays a role | No association between depression and social network measured |
| Husaini et al., 1990 | Social support and depression among the Black and White elderly | No structural measure of social network |
| Jang et al., 2010 | Correlates of Depressive Symptoms Among Hispanic Older Adults Living in Public Housing | Institutionalized population/ no separate analysis for community-dwelling population |
| Jang et al., 2016 | Emotional Confidants in Ethnic Communities: Social Network Analysis of Korean American Older Adults | No association between depression and social network measured |
| Jang et al., 2021 | Health risks posed by social and linguistic isolation in older Korean Americans | No measure of depression |
| Jayakody et al., 2022 | Is There an Association Between Untreated Hearing Loss and Psychosocial Outcomes? | No association between depression and social network measured |
| Jeon et al., 2016 | The Influence of Social Networks and Social Support on Health Among Older Koreans at High Risk of Depression | No association between depression and social network measured |
| Kabo et al., 2019 | A Social Relations and Networks Perspective of Depressive Symptoms in Older African Americans Relative to Two Other Ethno-racial Groups | Non-matching age restrictions |
| Katsumata et al., 2005 | Gender differences in the contributions of risk factors to depressive symptoms among the elderly persons dwelling in a community Japan | No association between depression and social network measured |
| Ke et al., 2019 | Social capital and the health of left-behind older adults in rural China: a cross-sectional study | No measure of depression |
| Killian & Turner, 2014 | Latent Class Typologies for Emotional Support Among Midlife and Aging Americans: Evidence from the National Health and Human Nutrition Examination Survey | No structural measure of social network |
| Kim & Jung, 2022 | Relational burden depression and loneliness among american older adults: An inquiry into the ‚Äòdark side of social capital‚Äô | No structural measure of social network |
| Kim et al., 2019 | Social Network Position Moderates the Relationship between Late-life Depressive Symptoms and Memory Differently in Men and Women | No association between depression and social network measured |
| Kotozaki et al., 2021 | Association between the social isolation and depressive symptoms after the great East Japan earthquake: findings from the baseline survey of the TMM CommCohort study | No association between depression and social network measured |
| Krause & Liang, 1993 | Stress social support and psychological distress among the Chinese elderly | No structural measure of social network |
| Krause, 1991 | Stress and isolation from close ties in later life | No structural measure of social network |
| Kuittinen et al., 2014 | Depressive Symptoms and Their Psychosocial Correlates Among Older Somali Refugees and Native Finns | No association between depression and social network measured |
| Lahdenperä et al., 2022 | Psychological Distress During the Retirement Transition and the Role of Psychosocial Working Conditions and Social Living Environment | No measure of depression |
| Lamar et al., 2022 | Social Engagement and All-Cause Mortality: A Focus on Participants of the Minority Aging Research Study | No association between depression and social network measured |
| Lau et al., 2019 | Social support network typologies and their association with dementia and depression among older adults in Singapore: a cross-sectional analysis | No measure of depression |
| Lebowitz et al., 2018 | Correlating Post-disaster Support Network Density with Reciprocal Support Relation Satisfaction: An Elderly Cohort Within One Year of the 2011 Japan Disasters | No structural measure of social network |
| Lebowitz et al., 2019 | Post-flood social support networks and morbidity in Joso City Japan | No structural measure of social network |
| Lee & Holm, 2011 | Family Relationships and Depression among Elderly Korean Immigrants | No structural measure of social network |
| Lee & Min, 2023 | Racial Differences in C-Reactive Protein, Depression Symptoms, and Social Relationships in Older Adults: A Moderated Network Analysis | No structural measure of social network |
| Lee et al., 2020 | Gender differences in social network of cognitive function among community-dwelling older adults | No association between depression and social network measured |
| Lee et al., 2022 | Association of social network properties with resilience and depression among community-based Korean population | Non-matching age restrictions |
| Lee et al., 2023 | Social integration and risk of mortality among African-Americans: the Jackson heart study | Non-matching age restrictions |
| Lee, 2021 | Different Discussion Partners and Their Effect on Depression among Older Adults | Non-matching age restrictions |
| Lei et al., 2016 | Social networks and health-related quality of life among Chinese old adults in urban areas: results from 4th National Household Health Survey | No measure of depression |
| Levula et al., 2018 | The Association Between Social Network Factors with Depression and Anxiety at Different Life Stages | No structural measure of social network |
| Li et al., 2013 | Social Support Resources and Post-Acute Recovery for Older Adults with Major Depression | Patient group |
| Lim et al., 2023 | Friendship in Later Life: A Pathway Between Volunteering Hours and Depressive Symptoms | No association between depression and social network measured |
| Litwin, 2010 | Social networks and well-being: a comparison of older people in Mediterranean and non-Mediterranean countries | No structural measure of social network |
| Liu et al., 2022 | Role of Multifaceted Social Relationships on the Association of Loneliness with Depression Symptoms: A Moderated Mediation Analysis | No measure of depression |
| Lohmann et al., 2023 | Social Mediators of the Association Between Depression and Falls Among Older Adults | No association between depression and social network measured |
| Loibl et al., 2022 | Worry about debt is related to social loneliness in older adults in the Netherlands | No association between depression and social network measured |
| Löwenstein & Frank, 2023 | Social Support Networks of Individuals with Depressive Disorders: A Cross-sectional Survey in Former Psychiatric Inpatients in Germany | Non-matching age restrictions |
| Luo & Li, 2023 | Trajectories of social isolation and depressive symptoms in mid- and later life: a parallel process latent growth curve analysis | No structural measure of social network |
| Luppa et al., 2012 | Natural course of depressive symptoms in late life. An 8-year population-based prospective study | Institutionalized population/ no separate analysis for community-dwelling population |
| Luppa et al., 2012 | Prevalence and risk factors of depressive symptoms in latest life - Results of the Leipzig Longitudinal Study of the Aged (LEILA 75+) | Institutionalized population/ no separate analysis for community-dwelling population |
| Ma et al., 2022 | Association between frailty and cognitive function in older Chinese people: A moderated mediation of social relationships and depressive symptoms | No association between depression and social network measured |
| Maity & Mukhopadhyay, 2015 | Social Support Social Network and Mental Health of Elderly: Rural-urban Differentials | No measure of depression |
| Mann & Walker, 2022 | The role of equanimity in mediating the relationship between psychological distress and social isolation during COVID-19 | Non-matching age restrictions |
| Mao & Chen, 2021 | Neighborhood-Based Social Capital and Depressive Symptoms among Adults: Evidence from Guangzhou China | Non-matching age restrictions |
| Masini & Barrett, 2008 | Social Support as a Predictor of Psychological and Physical Well-Being and Lifestyle in Lesbian Gay and Bisexual Adults Aged 50 and Over | Non-matching age restrictions |
| Maulik et al., 2010 | The effect of social networks and social support on common mental disorders following specific life events | No association between depression and social network measured |
| McHugh & Lawlor, 2012 | Social support differentially moderates the impact of neuroticism and extraversion on mental wellbeing among community-dwelling older adults | No association between depression and social network measured |
| Mechakra-Tahiri et al., 2009 | Social relationships and depression among people 65 years and over living in rural and urban areas of Quebec | No structural measure of social network |
| Meyer et al., 2022 | Neighborhood Characteristics and Caregiver Depressive Symptoms in the National Study of Caregiving | Non-matching age restrictions |
| Miller et al., 2006 | Feeling Blue? The Importance of a Confidant for the Well-Being of Older Rural Married Australian and American Men | No measure of depression |
| Milton et a., 2023 | Family of origin, not chosen family, predicts psychological health in a LGBTQ+ sample | Non-matching age restrictions |
| Monserud & Wong, 2015 | Depressive Symptoms Among Older Mexicans: The Role of Widowhood Gender and Social Integration | No structural measure of social network |
| Morita et al., 2022 | Depressive symptoms homophily among community-dwelling older adults in japan: A social networks analysis | No structural measure of social network |
| Myagmarjav et al., 2019 | Comparison of the 18-item and 6-item Lubben Social Network Scales with community-dwelling older adults in Mongolia | No association between depression and social network measured |
| Na & Streim, 2017 | Psychosocial Well-Being Associated With Activity of Daily Living Stages Among Community-Dwelling Older Adults | No association between depression and social network measured |
| Nadimpalli et al., 2015 | The Association Between Discrimination and Depressive Symptoms Among Older African Americans: The Role of Psychological and Social Factors | No association between depression and social network measured |
| Narendran et al., 2023 | Loneliness, social support networks, mood, and well-being among the community-dwelling elderly, Mysore | No structural measure of social network |
| Nyqvist et al., 2006 | Social Capital and Health in the Oldest Old: The Umea 85+ Study | No structural measure of social network |
| Osborn et al., 2003 | Factors associated with depression in a representative sample of 14 217 people aged 75 and over in the United Kingdom: results from the MRC trial of assessment and management of older people in the community | No structural measure of social network |
| Pan & Liu, 2021 | Difference of depression between widowed and non-widowed older people in China: A network analysis approach | No structural measure of social network |
| Panes et al., 2023 | Predictors of loneliness onset and maintenance in European older adults during the COVID-19 pandemic | No association between depression and social network measured |
| Park et al., 2015 | An empirical typology of social networks and its association with physical and mental health: a study with older Korean immigrants | No association between depression and social network measured |
| Park et al., 2020 | A Typology of Social Networks and Its Relationship to Psychological Well-Being in Korean Adults | Non-matching age restrictions |
| Pengpid & Peltzer, 2023 | Prevalence and correlates of major depressive disorder among a national sample of middle-aged and older adults in India | No structural measure of social network |
| Phongtankuel, 2023 | The relationship of caregiver self-efficacy to caregiver outcomes: a correlation and mediation analysis | Non-matching age restrictions |
| Ramos-Vera et al., 2023 | Psychological impact of COVID-19: A cross-lagged network analysis from the English Longitudinal Study of Aging COVID-19 database | Different scope |
| Rico-Uribe et al., 2016 | Loneliness Social Networks and Health: A Cross-Sectional Study in Three Countries | Non-matching age restrictions |
| Roberts et al., 1994 | Physical, Psychological, and Social Resources As Moderators of the Relationship of Stress to Mental Health of the Very Old | No measure of depression |
| Robinson & Austin, 1998 | Wife caregivers' and supportive others' perceptions of the caregivers' health and social support | No structural measure of social network |
| Roh et al., 2015 | Friends Depressive Symptoms and Life Satisfaction Among Older Korean Americans | Not adjusted for confounders |
| Rudert & Janke, 2023 | Call me maybe: Risk factors of impaired social contact during the COVID‐19 pandemic and associations with well‐being | Non-matching age restrictions |
| Ryu et al., 2022 | Impact of COVID-19 on the social relationships and mental health of older adults living alone: A two-year prospective cohort study | No association between depression and social network measured |
| Sahoo et al., 2022 | Depression and quality of life among elderly: Comparative cross-sectional study between elderly in community and old age homes in Eastern India | Institutionalized population/ no separate analysis for community-dwelling population |
| Sakurai et al., 2019 | Poor Social Network Not Living Alone Is Associated With Incidence of Adverse Health Outcomes in Older Adults | No association between depression and social network measured |
| Sakurai et al., 2021 | Association of Eating Alone With Depression Among Older Adults Living Alone: Role of Poor Social Networks | No association between depression and social network measured |
| Salazar et al., 2022 | Risk factors for depression in older adults in Bogotá, Colombia. | No structural measure of social network |
| Santini et al., 2020 | Social disconnectedness perceived isolation and symptoms of depression and anxiety among older Americans (NSHAP): a longitudinal mediation analysis | No measure of depression |
| Sasiwongsaroj et al., 2015 | Buddhist social networks and health in old age: A study in central Thailand | No structural measure of social network |
| Savela et al., 2022 | Addressing the Experiences of Family Caregivers of Older Adults During the COVID-19 Pandemic in Finland | No structural measure of social network |
| Schaefer et al., 1981 | The health-related functions of social support | Non-matching age restrictions |
| Schnittger et al., 2012 | Psychological distress as a key component of psychosocial functioning in community-dwelling older people | No association between depression and social network measured |
| Schutter et al., 2020 | 'Big Five' personality characteristics are associated with loneliness but not with social network size in older adults irrespective of depression | No association between depression and social network measured |
| Schwartz & Litwin, 2019 | The Reciprocal Relationship Between Social Connectedness and Mental Health Among Older European Adults: A SHARE-Based Analysis | No association between depression and social network measured |
| Schwartz et al., 2019 | Contact frequency and cognitive health among older adults in Israel | No structural measure of social network |
| Segrin, 2003 | Age Moderates the Relationship between Social Support and Psychosocial Problems | Non-matching age restrictions |
| Shahaj et al., 2023 | Psychological Distress Among Older Adults During the First Wave of SARS-CoV-2 Pandemic: Survey of Health, Ageing, and Retirement in Europe | No measure of depression |
| Sharma et al., 2023 | Does emotion regulation network mediate the effect of social network on psychological distress among older adults? | No measure of depression |
| Shou et al., 2018 | Quality of life and its contributing factors in an elderly community-dwelling population in Shanghai China | No association between depression and social network measured |
| Shrum et al., 2021 | The Burden of Elders Anxiety Depression and Personal Networks in Two African Slums | Non-matching age restrictions |
| Simning et al., 2012 | Mental healthcare need and service utilization in older adults living in public housing | No measure of depression |
| Steffens et al., 2005 | Biological and social predictors of long-term geriatric depression outcome | No association between depression and social network measured |
| Stewart et al., 2022 | Functional and structural social support in DSM-5 mood and anxiety disorders: A population-based study | Non-matching age restrictions |
| Stokes et al., 2018 | Influence of the Social Network on Married and Unmarried Older Adults' Mental Health | No association between depression and social network measured |
| Sugisawa & Sugihara, 2020 | Mediators and Moderators of the Influences of Living Alone on Psychological Distress Among Japanese Older Adults | No measure of depression |
| Sugisawa et al., 2022 | Mediators of Life-Course and Late-Life Financial Strain on Late-Life Health in Japan: Based on a Cross-Sectional Survey | No structural measure of social network |
| Sunderland et al., 2014 | Comparing profiles of mental disorder across birth cohorts: Results from the 2007 Australian National Survey of Mental Health and Wellbeing | No structural measure of social network |
| Tang et al., 2023 | Residential Segregation and Depressive Symptoms in Older Chinese Immigrants: The Mediating Role of Social Processes | No access to full text (first author has been contacted) |
| Thiyagarajan et al., 2014 | Social support network typologies and health outcomes of older people in low and middle income countries--a 10/66 Dementia Research Group population-based study | Institutionalized population/ no separate analysis for community-dwelling population |
| Thomas, 2016 | The Impact of Relationship-Specific Support and Strain on Depressive Symptoms Across the Life Course | No structural measure of social network |
| Tiedt, 2010 | The gender gap in depressive symptoms among Japanese elders: evaluating social support and health as mediating factors | No structural measure of social network |
| Tinghog et al., 2010 | The Association of Immigrant- and Non-Immigrant-Specific Factors With Mental Ill Health Among Immigrants in Sweden | Non-matching age restrictions |
| Triolo et al., 2020 | Social engagement in late life may attenuate the burden of depressive symptoms due to financial strain in childhood | No association between depression and social network measured |
| Triolo et al., 2022 | Pre-pandemic Physical Function and Social Network in Relation to COVID-19-Associated Depressive Burden in Older Adults in Sweden | No structural measure of social network |
| Tucker et al., 2022 | Marital Transitions, Change in Depressive Symptomology, and Quality of Social Relationships in Midlife and Older U.S. Adults: An Analysis of the Health and Retirement Study | No structural measure of social network |
| van Beljouw et al., 2014 | "Being all alone makes me sad": loneliness in older adults with depressive symptoms | No association between depression and social network measured |
| van den Brink et al., 2018 | Prognostic significance of social network social support and loneliness for course of major depressive disorder in adulthood and old age | Non-matching age restrictions |
| Vancampfort et al., 2020 | Sedentary behavior and depression among community-dwelling adults aged >= 50 years: Results from the irish longitudinal study on Ageing | No association between depression and social network measured |
| Villamil et al., 2006 | Low Prevalence of Depression and Anxiety Is Linked to Statutory Retirement Ages Rather than Personal Work Exit: A National Survey | No structural measure of social network |
| Wahlin et al., 2015 | Prevalence of depressive symptoms and suicidal thoughts among elderly persons in rural Bangladesh | No structural measure of social network |
| Wallsten et al., 1999 | Disability and depressive symptoms in the elderly: The effects of instrumental support and its subjective appraisal | No structural measure of social network |
| Wang et al., 2023 | Economic development, weak ties, and depression: Evidence from China | Non-matching age restrictions |
| Watanabe et al., 2004 | Social support and depressive symptoms among displaced older adults following the 1999 Taiwan earthquake | No structural measure of social network |
| Weitzer et al., 2022 | Dispositional optimism and depression risk in older women in the Nurses ' Health Study: a prospective cohort study | No association between depression and social network measured |
| Werner-Seidler et al., 2017 | The relationship between social support networks and depression in the 2007 National Survey of Mental Health and Well-being | No mean age |
| Wilby, 2011 | Depression and social networks in community dwelling elders: a descriptive study | No association between depression and social network measured |
| Williams et al., 1995 | Identifying depressive symptoms among elderly Medicare HMO enrollees | No access to full text (first author has been contacted) |
| Wojszel & Politynska, 2021 | The structure and functional correlates of social support networks of people in advanced old age living in chosen urban and rural areas in Poland: a cross‑sectional study | Not adjusted for confounders |
| Won et al., 2021 | The mediating effect of life satisfaction and the moderated mediating effect of social support on the relationship between depression and suicidal behavior among older adults | No structural measure of social network |
| Woo et al., 1994 | The prevalence of depressive symptoms and predisposing factors in an elderly Chinese population | Institutionalized population/ no separate analysis for community-dwelling population |
| Wu et al., 2018 | Network-based and cohesion-based social capital and variations in depressive symptoms among Taiwanese adults | Non-matching age restrictions |
| Xiong et al., 2023 | The Relationship between Physical Activity and Mental Depression in Older Adults during the Prevention and Control of COVID-19: A Mixed Model with Mediating and Moderating Effects | No association between depression and social network measured |
| Yao et al., 2008 | Relationships between personal depression and social network factors and sleep quality in community-dwelling older adults | No association between depression and social network measured |
| Yu & Mahendran, 2021 | COVID-19 lockdown has altered the dynamics between affective symptoms and social isolation among older adults: results from a longitudinal network analysis | No structural measure of social network |
| Yu et al., 2023 | Social network and mental health of chinese immigrants in affordable senior housing during the covid-19 pandemic: A mixed-methods study | Qualitative |
| Zeng et al., 2013 | Family and social aspects associated with depression among older persons in a Chinese context | No association between depression and social network measured |
| Zhang & Chen, 2022 | Association between workplace and mental health and its mechanisms during COVID-19 pandemic: A cross-sectional, population-based, multi-country study | Non-matching age restrictions |
| Zhou et al., 2022 | Association between social capital and depression among older adults of different genders: Evidence from Hangzhou, China | No structural measure of social network |
| Zwar et al., 2023 | Mental health, social integration and support of informal caregivers during the second wave of the COVID-19 pandemic: A population-based representative study from Germany | Non-matching age restrictions |

Table A3 Quality appraisal: Newcastle-Ottawa-Scale (NOS) for cross-sectional studies

| **Author, Year** | **Selection** | **Comparability** | **Outcome** | **Evaluation** |
| --- | --- | --- | --- | --- |
| Ali et al., 2022 | 3 | 2 | 2 | *Good* |
| Antonucci et al., 1997 | 4 | 2 | 2 | *Good* |
| Aung et al., 2016 | 3 | 2 | 2 | *Good* |
| Bae et al., 2020 | 3 | 2 | 2 | *Good* |
| Becker et al., 2019 | 2 | 2 | 1 | *Poor* |
| Bincy et al., 2022 | 4 | 2 | 2 | *Good* |
| Bisconti & Bergeman, 1999 | 1 | 1 | 1 | *Poor* |
| Blumstein et al., 2004 | 3 | 2 | 1 | *Poor* |
| Boey & Chiu, 2005 | 4 | 2 | 2 | *Good* |
| Braam et al., 1997 | 4 | 2 | 2 | *Good* |
| Cao et al., 2015 | 3 | 2 | 2 | *Good* |
| Castro-Costa et al., 2008 | 0 | 1 | 2 | *Poor* |
| Chan & Zeng, 2009 | 5 | 2 | 2 | *Good* |
| Chan & Zeng, 2011 | 4 | 2 | 2 | *Good* |
| Chan et al., 2011 | 4 | 2 | 2 | *Good* |
| Cheng et al., 2014 | 3 | 2 | 1 | *Poor* |
| Chi & Chou, 2001 | 3 | 2 | 2 | *Good* |
| Cho et al., 2018 | 4 | 2 | 2 | *Good* |
| Choi & Jeon, 2021 | 3 | 2 | 2 | *Good* |
| Chou & Chi, 2001 | 4 | 2 | 2 | *Good* |
| Domènech-Abella et al., 2017 | 3 | 2 | 2 | *Good* |
| Dorrance Hall et al., 2019 | 3 | 2 | 2 | *Good* |
| Ermer & Proulx, 2022 | 2 | 2 | 2 | *Fair* |
| Fernández & Rosell, 2022 | 4 | 2 | 2 | *Good* |
| Fiori et al., 2006 | 3 | 2 | 2 | *Good* |
| Forsman et al., 2012 | 4 | 2 | 2 | *Good* |
| Frediksen-Goldsen et al., 2013 | 3 | 2 | 2 | *Good* |
| Fuller-Iglesias et al., 2008 | 4 | 2 | 1 | *Poor* |
| Gao et al., 2022 | 4 | 2 | 2 | *Good* |
| Goldberg et al., 1985 | 3 | 1 | 2 | *Good* |
| Golden et al., 2009 | 4 | 2 | 2 | *Good* |
| Gu et al., 2023 | 3 | 2 | 2 | *Good* |
| Gumà & Fernández-Carro, 2021 | 3 | 2 | 2 | *Good* |
| Hamid et al., 2019 | 3 | 1 | 2 | *Good* |
| Han et al., 2007 | 2 | 2 | 2 | *Fair* |
| Harada et al., 2023 | 3 | 2 | 2 | *Good* |
| Harasemiw et al., 2019 | 3 | 2 | 2 | *Good* |
| Jang et al., 2002 | 4 | 2 | 2 | *Good* |
| Jang et al., 2011 | 2 | 2 | 2 | *Fair* |
| Jeon & Lubben, 2016 | 2 | 1 | 2 | *Fair* |
| Jiang et al., 2022 | 3 | 2 | 2 | *Good* |
| Kim & Lee, 2015 | 4 | 2 | 2 | *Good* |
| Kim & Lee, 2019 | 2 | 2 | 2 | *Fair* |
| Kim et al., 2012 | 3 | 2 | 2 | *Good* |
| Kim et al., 2015 | 2 | 2 | 2 | *Fair* |
| Klug et al., 2014 | 5 | 2 | 2 | *Good* |
| La Gory & Fitpatrick, 1992 | 2 | 2 | 1 | *Poor* |
| Lee & Chou, 2019 | 3 | 2 | 2 | *Good* |
| Lee et al., 1996 | 2 | 1 | 1 | *Poor* |
| Lee et al., 2017 | 3 | 2 | 2 | *Good* |
| Li et al., 2019 | 2 | 2 | 2 | *Fair* |
| Li et al., 2022 | 4 | 2 | 2 | *Good* |
| Litwin & Levinsky, 2022 | 3 | 2 | 2 | *Good* |
| Litwin & Levinsky, 2023 | 3 | 2 | 2 | *Good* |
| Litwin et al., 2015 | 4 | 2 | 2 | *Good* |
| Litwin, 2011 | 2 | 2 | 2 | *Fair* |
| Litwin, 2012 | 2 | 2 | 2 | *Fair* |
| Liu et al., 2016 | 1 | 2 | 2 | *Poor* |
| Marshall & Rue, 2012 | 3 | 2 | 2 | *Good* |
| Marshall-Fabien & Miller, 2016 | 3 | 2 | 2 | *Good* |
| Mechakra-Tahiri et al., 2010 | 4 | 2 | 2 | *Good* |
| Merchant et al., 2020 | 2 | 1 | 2 | *Fair* |
| Merhabi & Béland, 2021 | 2 | 2 | 2 | *Fair* |
| Miller & Lago, 1990 | 3 | 1 | 1 | *Poor* |
| Minicuci et al., 2002 | 3 | 2 | 2 | *Good* |
| Murayama et al., 2014 | 2 | 2 | 2 | *Fair* |
| Okwumabua et al., 1997 | 3 | 2 | 1 | *Poor* |
| Palinkas et al., 1990 | 1 | 2 | 2 | *Poor* |
| Park & Roh, 2013 | 3 | 2 | 2 | *Good* |
| Park et al., 2013 | 3 | 2 | 2 | *Good* |
| Park et al., 2014 | 2 | 2 | 2 | *Fair* |
| Park et al., 2018 | 3 | 2 | 2 | *Good* |
| Park et al., 2019 | 3 | 2 | 2 | *Good* |
| Pavlidis et al., 2023 | 2 | 2 | 2 | *Fair* |
| Pilehvari et al., 2023 | 3 | 2 | 2 | *Good* |
| Roh et al., 2015 | 3 | 2 | 2 | *Good* |
| Santini et al., 2015 | 4 | 2 | 2 | *Good* |
| Shouse et al., 2013 | 2 | 1 | 2 | *Fair* |
| Sicotte et al., 2008 | 3 | 2 | 2 | *Good* |
| Singh et al., 2016 | 2 | 2 | 2 | *Fair* |
| Sohn et al., 2017 | 3 | 2 | 2 | *Good* |
| Sonnenberg et al., 2013 | 4 | 2 | 2 | *Good* |
| Stoeckel & Litwin, 2016 | 2 | 2 | 2 | *Fair* |
| Sugie et al., 2022 | 3 | 2 | 2 | *Good* |
| Tang & Xie, 2021 | 4 | 2 | 2 | *Good* |
| Tang et al., 2020 | 5 | 2 | 2 | *Good* |
| Tang et al., 2023 | 4 | 2 | 2 | *Good* |
| Tanikaga et al., 2023 | 3 | 2 | 2 | *Good* |
| Taylor et al., 2018 | 3 | 2 | 2 | *Good* |
| Taylor, 2021 | 3 | 2 | 2 | *Good* |
| Tsai et al., 2005 | 3 | 1 | 2 | *Good* |
| Vicente & Guadalupe, 2022 | 1 | 2 | 2 | *Poor* |
| Webster et al., 2015 | 2 | 2 | 2 | *Fair* |
| Wee et al., 2014 | 2 | 2 | 2 | *Fair* |
| Wendel et al., 2022 | 3 | 2 | 2 | *Good* |
| Wu et al., 2017 | 3 | 2 | 2 | *Good* |
| Ye & Zhang, 2019 | 2 | 2 | 2 | *Fair* |
| *Threshold for converting the NOS for cross-sectional studies: good quality (3 to 5 stars in selection domain AND 1 or 2 stars in comparability domain AND 2 or 3 stars in outcome domain), fair quality (2 stars in selection domain AND 1 or 2 stars in comparability domain AND 2 or 3 stars in outcome domain), poor quality (0 or 1 star in selection domain OR 0 stars in comparability domain OR 0 or 1 stars in outcome domain)* | | | | |

Table A4 Quality appraisal: Newcastle-Ottawa-Scale (NOS) for longitudinal studies

| **Author, Year** | **Selection** | **Comparability** | **Outcome** | **Evaluation** |
| --- | --- | --- | --- | --- |
| Bisschop et al., 2004 | 3 | 2 | 3 | *Good* |
| Blumstein et al., 2004 | 3 | 2 | 2 | *Good* |
| Bui, 2020 | 4 | 2 | 2 | *Good* |
| Byers et al., 2012 | 4 | 1 | 2 | *Good* |
| Chao, 2011 | 3 | 2 | 3 | *Good* |
| Coleman et al., 2022 | 3 | 2 | 2 | *Good* |
| Domènech-Abella et al., 2019 | 4 | 2 | 3 | *Good* |
| Förster et al., 2018 | 3 | 2 | 2 | *Good* |
| Förster et al., 2021 | 4 | 2 | 2 | *Good* |
| Gan & Best, 2021 | 2 | 2 | 2 | *Fair* |
| Hajek & König, 2016 | 3 | 2 | 2 | *Good* |
| Harlow et al., 1991 | 2 | 1 | 3 | *Fair* |
| Holwerda et al., 2023 | 3 | 2 | 2 | *Good* |
| Houtjes et al., 2014 | 3 | 2 | 3 | *Good* |
| Husaini, 1997 | 2 | 0 | 1 | *Poor* |
| Kim et al., 2016 | 3 | 2 | 2 | *Good* |
| Kuchibhatla et al., 2012 | 3 | 2 | 3 | *Good* |
| Litwin & Levinsky, 2021 | 2 | 2 | 2 | *Fair* |
| Litwin et al., 2020 | 2 | 2 | 2 | *Fair* |
| Oxman et al., 1992 | 2 | 0 | 3 | *Poor* |
| Reynolds et al., 2020 | 3 | 2 | 3 | *Good* |
| Ruan et al., 2022 | 4 | 2 | 3 | *Good* |
| Santini et al., 2016 | 4 | 2 | 2 | *Good* |
| Santini et al., 2017 | 3 | 2 | 2 | *Good* |
| Santini et al., 2021 | 2 | 1 | 2 | *Fair* |
| Schwartz & Litwin, 2017 | 3 | 2 | 3 | *Good* |
| Stringa et al., 2020 | 2 | 1 | 2 | *Fair* |
| Tang et al., 2023 | 3 | 2 | 2 | *Good* |
| Voils et al., 2007 | 2 | 1 | 3 | *Fair* |
| Werneck et al., 2023 | 4 | 2 | 2 | *Good* |
| Zhang et al., 2023 | 4 | 2 | 3 | *Good* |
| *Threshold for converting the NOS for longitudinal studies: good quality (3 to 4 stars in selection domain AND 1 or 2 stars in comparability domain AND 2 or 3 stars in outcome domain), fair quality (2 stars in selection domain AND 1 or 2 stars in comparability domain AND 2 or 3 stars in outcome domain), poor quality (0 or 1 star in selection domain OR 0 stars in comparability domain OR 0 or 1 stars in outcome domain)* | | | | |
